# Biomimetic Versatile Anisotropic, Electroactive Cellulose Hydrogel Scaffolds Tailored from Fern Stem Serving as Nerve Conduit and Cardiac Patch

**DOI:** 10.1002/advs.202400002

**Published:** 2024-12-04

**Authors:** Qinghui Liang, Shuhui Chen, Shaofeng Hua, Weihong Jiang, Jiamian Zhan, Chunyi Pu, Rurong Lin, Yutong He, Honghao Hou, Xiaozhong Qiu

**Affiliations:** ^1^ Department of Anatomy Guangdong Provincial Key Laboratory of Construction and Detection in Tissue Engineering School of Basic Medical Sciences Southern Medical University Guangzhou Guangdong 510515 P. R. China

**Keywords:** anisotropic, cellulose, *Dicranopteris linearis*, electroactive, engineered cardiac patch, nerve guidance conduit

## Abstract

Peripheral nerve injury (PNI) and myocardial infarction (MI) are the two most clinically common soft excitable tissue injuries. Both nerve and cardiac tissues exhibit structural anisotropy and electrophysiological activity, providing a wide range of biophysical cues for cell and tissue repair. However, balancing microstructural anisotropy, electroactivity, and biocompatibility is challenging. To address this issue, *Dicranopteris linearis* (*D. linearis*) is proposed as a low‐perceived value fern plant. Moreover, to enhance its usefulness, it can be designed into a tubular structure and a lamellar structure to bridge the damaged tissue. Therefore, a robust yet simple top‐down approach is proposed to designing and fabricating the desired biomimetic versatile hydrogels orienting from the *D. linearis* to customize for different soft excitable tissue repair applications. These anisotropic electroactive hydrogels performed well as nerve guidance conduits (NGC) and engineered cardiac patches (ECP) in the repair of PNI and MI, respectively. Two birds, one stone. Accordingly, the biomimetic strategy of *D. linearis* to NGC and *D. linearis* to ECP is first proposed, opening a new horizon for constructing tissue engineering using natural sources.

## Graphical abstract

Inspired by the naturally anisotropic structure of soft excitable tissue, a robust yet simple top‐down approach is proposed to fabricating the desired biomimetic versatile hydrogels orienting from the *Dicranopteris linearis*. Two birds, one stone. These anisotropic electroactive hydrogels performed well as nerve guidance conduits and engineered cardiac patches in the repair of peripheral nerve injury and myocardial infarction, respectively.

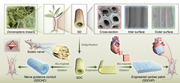

## 1 Introduction

Tissue dysfunction or organ loss caused by aging, severe trauma, or diseases results in the loss of proper tissue function and imposes medically and economically significant burdens on the patient community and society. For example, peripheral nerve injury (PNI) and myocardial infarction (MI) are two most clinically common soft excitable tissue injuries in the nervous system and the cardiovascular system, respectively.^[^ ^1^ ^]^ PNI is a seriously disabling chronic neurosurgical illness caused primarily by acute trauma, with over 5 million new cases diagnosed each year around the world.^[^ ^2^ ^]^ According to the Global Burden of Disease study, ischemic cardiovascular diseases, represented by MI, is the leading cause of death worldwide, with an estimated 18.6 million prevalent cases in 2019.^[^ ^3^ ^]^ Given the limited therapeutic effect of currently available clinical approaches, tissue‐engineered biological scaffolds or patches have emerged as a promising strategy for tissue injury repair and regeneration, capable of providing the appropriate structural and physiological microenvironment as well as acting as an active modulator for endogenous cells and injured tissue.^[^ ^1^, ^4^ ^]^ Despite the fact that a large number of engineered biomaterials have been designed and tested in vivo and in vitro, the design and fabrication of versatile biomaterials through robust and simple preparation processes to meet the needs of various soft tissues remains a significant challenge.^[^ ^5^ ^]^

The capabilities to mimic the biophysical and chemical characteristics of the targeted injured tissues in vivo extracellular matrix (ECM) are still finite and deficient. The nerve tract and myocardial fiber bundle both exhibit structural anisotropy and electrophysiological activity, providing a wide range of biophysical and biochemical cues for cell and tissue repair (Figure S1, Supporting Information).^[^ ^6^ ^]^ First, structural anisotropy governs the orientation of filaments and cells, consequently influencing anisotropic tissue properties. Biomimetic anisotropic structures, which are inspired by ECM and oriented tissues of biological soft tissues, play an important role in the design of engineered biomaterial hydrogels for soft tissue repair.^[^ ^7^ ^]^ A number of novel approaches and techniques (pre‐stretching, external field control, 3D printing, and electrostatic spinning) were proposed to construct biomimetic anisotropic hydrogels that mimic ECM, allowing them better to mimic the mechanical and functional properties of target tissues.^[^ ^8^ ^]^ Furthermore, the nerve and cardiac tissues exhibit electrically excited activity.^[^ ^5^, ^9^ ^]^ Specifically, their electrophysiological activities must be mutually affected by these electrical microenvironment changes in cells and/or tissues.^[^ ^6b^ ^]^ Remodeling the mechanical‐electro coupling microenvironment is promising for restoring well‐established electrical conduction between the injured area and normal tissues, resulting in unhindered electrophysiological activity and functions.^[^ ^10^ ^]^ The tubulose structure with electroactivity, for example, was used to bridge the sciatic nerve gap and transmit biomimetic bioelectric signals.^[^ ^11^ ^]^ Similarly, the electroactive lamellar structure was used to bridge healthy myocardium across the scar region in order to restore the conductive microenvironment.^[^ ^5b^ ^]^ Therefore, replicating these biophysical cues of the ECM using biomimetic anisotropic scaffolds represents great importance. Conversely, although much progress has been made, balancing microstructural anisotropy, electroactivity, and biocompatibility remains a challenge.^[^ ^12^ ^]^ To design novel anisotropic and electroactive hydrogels, different natural and synthetic biomaterials were combined using various fabrication technologies to create versatile tissue engineering scaffolds. However, several synthetic polymers may cause unwanted inflammation, resulting in the formation of undesired tissue, and natural materials are limited by inherent properties such as bulkiness, low mechanical properties, non‐transparency, and nonconductivity.^[^ ^13^ ^]^ The processing of novel approaches and techniques is time‐consuming and costly.^[^ ^14^ ^]^ Furthermore, an irreconcilable trade‐off usually exists between the biocompatibility and versatility of these biomimetic scaffolds.^[^ ^23^ ^]^

Recently, many researchers have concentrated their efforts on wood‐based materials, which are prime examples of the excellent hierarchical anisotropic structure found in plants.^[^ ^13^, ^15^ ^]^ Their abundance, low cost, good biocompatibility, environmental friendliness, and degradability far outweigh those of synthetic polymers and other biomaterials.^[^ ^16^ ^]^ Owing to their excellent properties and advantages, they have been designed into various biomimetic anisotropic hydrogels and have been used in a wide range of applications.^[^ ^17^ ^]^ The advancement and investigation of the functions of wood‐based materials may lead to increased demand, potentially resulting in the overexploitation of wood from trees with longer growth periods. In contrast, we propose *Dicranopteris linearis* (*D. linearis*) as an alternative option for constructing biomimetic anisotropic hydrogels. *D. linearis* is a common fern in Southeast Asia with a low‐perceived value that is frequently undervalued and neglected, resulting in it being discarded as waste. Biomimetic anisotropic hydrogels were prepared from the stem of *D. linearis* (SD), which has a unique tubular structure consisting of a cortex composed of multiple layers of parenchyma cells, to make better use of waste *D. linearis*. When scaffold materials are prepared to meet the requirements of tissue repair, the SD can be designed and manipulated to maintain a tubular structure, expand into a lamellar structure, or construct into a complex structure.^[^ ^18^ ^]^ During this process, the top‐down approach is more applicable to transforming SD into functional materials compared to the traditional bottom‐up approach, which involves a complex multistep manufacturing process with unsatisfactory manufacturing efficiency.^[^ ^19^ ^]^

**Scheme 1:**
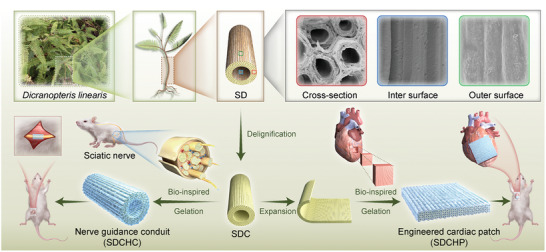
Schematic illustration of the manufacturing process and bioapplications of biomimetic versatile anisotropic, electroactive cellulose hydrogel scaffolds orienting from the *Dicranopteris linearis* served as nerve guidance conduit (NGC) and engineered cardiac patch (ECP) based on the natural anisotropic structure of nerve tract and myocardial fiber bundle.

In this work, as shown in **Scheme** **1**, we report a robust yet simple top‐down approach to design and customize the desired biomimetic anisotropic electroactive hydrogels oriented from the plant‐resourced SD for different soft excitable tissue repair applications.^[^ ^20^ ^]^ We demonstrated the adaptability and designability of anisotropic electroactive hydrogel scaffolds as biomedical conduits or patches for a variety of in vivo applications. First, we tailored the originally stiff, hydrophobic, and nonconductive SD to the SD‐derived cellulose scaffold (termed SDC) through delignification, exhibiting a structure similar to that of natural soft excitable tissue. By in situ polymerizing conductive polyacrylic acid (PAA, FDA‐approved) or PAA/GelMa, we engineered the SDC into a flexible, electroactive, and anisotropic hydrogel. Surprisingly, the anisotropic structure of the SDC was largely preserved after simple preparation, which is critical for mimicking the specific ECM‐like architecture of soft tissues. In order to address various bioapplication scenarios for soft tissue repair, biomimetic anisotropic electroactive hydrogels such as the SD‐derived cellulose hydrogel conduit (SDCHC) for PNI and the SD‐derived cellulose hydrogel patch (SDCHP) for MI were created. These hydrogels possess desirable properties such as biocompatibility, anisotropy, electroactivity, mechanical durability, and permeability, which allow them to benefit cell functionalization and promote tissue repair performance. Two birds, one stone. The biomimetic strategy of “*D. linearis* to nerve guidance conduit (NGC)” and “*D. linearis* to engineered cardiac patch (ECP)” was first proposed, opening a new horizon for constructing tissue engineering using natural sources.

## 2 Results and Discussion

### 2.1 Design and Fabrication of Biomimetic Anisotropic and Electroactive SDCHC

**Figure 1:**
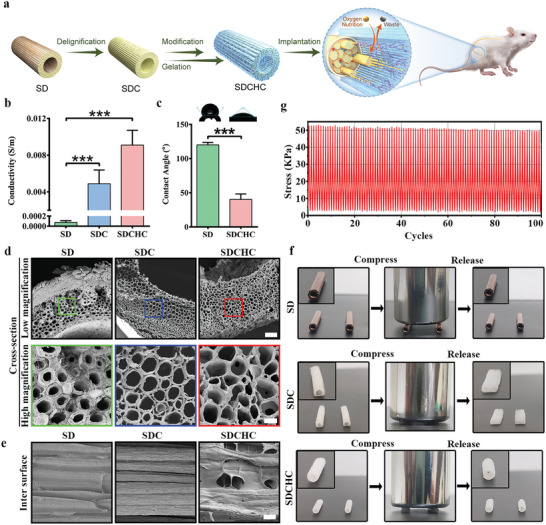
Fabrication and characterization of the SDCHC. a) Schematic of the construction and application of the SDCHC for PNI. b) Analysis of electrical conductivity of the SD, SDC, and SDCHC (data are presented as mean ± S.D. *p < 0.05; **p < 0.01; ***p < 0.001. *p*‐value was generated by ANOVA and Tukey's test, *n* = 5 independent samples). c) Analysis of contact angle of the SD and SDCHC (Data were presented as mean ± S.D. *p < 0.05; **p < 0.01; ***p < 0.001. unpaired *t*‐tests were used for comparisons, *n* = 4 independent samples). d) SEM image of the cross‐sections in the SD, SDC, and SDCHC. Scale bars: 100 µm at low magnification; 20 µm at high magnification. e) SEM image of the intersurface in the SD, SDC, and SDCHC. Scale bars: 20 µm. f) Comparison before and after 100 g weight compressed the SD, SDC, and SDCHC. g) Cyclic compression testing of the SDCHC at 30% strain up to 100 cycles.

At the present time, balancing microstructural anisotropy, electroactivity, and biocompatibility is challenging. The plant‐resourced SD stands out due to its abundance, low cost, good biocompatibility, eco‐friendliness, and unique tubulose structure, rendering it an ideal material to address this issue. In the preparation of the scaffold, the SD tubulose structure was chosen as the raw natural biomaterial for PNI repair, which was adopted to bridge the sciatic nerve gap. However, its originally stiff, hydrophobic, and nonconductive properties limited the promoting effect of nerve regeneration. Therefore, we propose the development of a versatile anisotropic, electroactive cellulose‐based hydrogel originating from SD to promote the regeneration and repair of nerves. To overcome the challenges, the SDC was extracted from the SD through delignification and then modification with vinyl trichlorosilane (VTC; **Figure** **1**a). During the delignification process using sodium hypochlorite, the lignin and hemicellulose components from the cell wall of the SD were selectively removed.^[^ ^21^ ^]^ Consequently, the SDC was obtained from the SD by dispersing the lignin in the bulk solution. Fourier transform‐infrared (FT‐IR) spectroscopy and energy‐dispersive X‐ray spectroscopy (EDS) spectrawere performed to confirm the compositional evolution of the SDC from SD. FT‐IR analysis of the SDC revealed that the peaks at 1735 and 1260 cm^−1^ of SDC almost disappeared with a significant decrease of peak intensities compared to that of SD, corresponding to the carboxyl groups of hemicellulose and the ester bond of the carboxyl groups of lignin and/or hemicellulose (Figure S2a, Supporting Information), respectively.^[^ ^18^ ^]^ This result indicates that lignin and hemicellulose contents in the SD were almost removed in the delignification process. Furthermore, the ELISA findings reveal that the content ratio of cellulose, hemicellulose, and lignin changed in the delignification process from the SD to SDC (Figure S3, Supporting Information), which is consistent with the results of FT‐IR.^[^ ^18^ ^]^

To obtain better properties and further functionalization of SDC, the SDC‐VTC with reactible ethene bonds was formed after being surface‐functionalized in the presence of VTC. The FT‐IR spectrum of SDC‐VTC demonstrated a notable increase at 1735 cm^−1^, which is associated with the functional vinyl groups of the VTC. EDS mapping images of SDC‐VTC revealed that C, O, and Si atoms were uniformly distributed on the surface of the SDC‐VTC, and the weight concentration of the Si atom was determined to be ≈1.1% (Figure S2b, Supporting Information).^[^ ^22^ ^]^ These characterizations verified the successful graft of VTC onto the SDC. Subsequently, we obtained the SDCHC with the desired biomimetic anisotropy and electroactivity by in situ polymerized acrylic acid and cross‐linking it on the surface of the SDC‐VTC composite. This biomimetic anisotropic hydrogel may serve as a foundation for future direct application as NGC in nerve tissue engineering.

### 2.2 Characterization of Biomimetic Anisotropic and Electroactive SDCHC

Accumulating evidence demonstrated that electroactive materials have emerged as a critical parameter to facilitate peripheral nerve regeneration by regulating cell behavior, signaling pathways, and immune microenvironment.^[^ ^23^ ^]^ The electrical conductivity of each conduit hydrogel material was measured using cyclic voltammetry (CV) and electrochemical impedance spectra (EIS). Figure 1b illustrates that the electrical conductivity of SDC was found to be (4.9 ± 1.5) × 10^−3^ S m^−1^, which was higher than that of nonconductive SD due to the dispersion of the lignin and the good orientation of the SDC.^[^ ^24^ ^]^ However, after the gel network was prepared, the electrical conductivity of SDCHC was found to be (9.1 ± 1.6) × 10^−3^ S m^−1^, which was higher than that of both SDC and nonconductive SD. Furthermore, the stability of the electrical conductivity and the EIS of SDCHC was tested in PBS at 37 °C for more than 1 week. The electrical conductivity and EIS value at 1000 Hz of SDCHC were stable at the same time points for more than 9 days (Figure S4a,c, Supporting Information). Then the long‐term stability of the electrical conductivity of SDCHC was checked at 1, 30, 90, and 180 days. It is found that these scaffold samples still maintained the robust stability of ionic conductivity over 6 months, demonstrating the feasibility and stability potential for long‐term in vivo tissue engineering (Figure S4b, Supporting Information).

Surface wettability is important for cell adhesion and further functionality. Thus, we determined the hydrophilicity of each conduit hydrogel material using an optical contact angle measuring instrument. Contact angles below 90° are hydrophilic, and those above 90° are hydrophobic. Moreover, Figure 1c demonstrates that the contact angle of SD was tested to be 120 ± 3.6°, which is hydrophobic. Following treatment, the contact angle of SDCHC decreased to 40 ± 8.1°, indicating that the SDCHC surface was converted to hydrophilic from the originally hydrophobic SD. The swelling ratio of each scaffold was estimated to characterize water absorption. The swelling ratio of SDCHC reached 1.6 times its initial weight after 2 min of swelling in PBS and maintained to a state of swollen equilibrium stably for more than 5 days (Figure S5a, Supporting Information). The swelling ratio should be ascribed to PBS, which can effectively diffuse into this hydrogel at a faster rate to form a porous structure.

In neural tissue engineering, a neural scaffold must have sufficient permeability for nutrient and oxygen exchange and to avoid pressure due to waste retention, which is directly related to the 3D porous microstructure. Scanning electron microscopy (SEM) was used to examine the morphology and porous microstructures of each scaffold. Figure 1d displays that there are many microchannels in the longitudinal direction in the cross‐section of SD and SD‐derived scaffolds (SDC, and SDCHC). These intrinsic microchannels preserved in ECM scaffolds play a vital role in promoting tissue ingrowth and vascularization upon in vivo implantation.^[^ ^25^ ^]^ Additionally, the luminal surface of SDC exhibited abundant pore structure and submicro structures running along the longitudinal direction of the channels compared to the SD (Figure S6, Supporting Information). On the surface of SDCHC, internal interconnected microporous structures and anisotropic microstructure were observed, facilitating the induction of cell‐oriented arrangement and exchange of oxygen and nutrients (Figure 1e).

The tubulose structure of the conduit in the manufacture of NGCs is difficult to be maintain and NGCs can easily collapse once they bore multiple mechanical deformations after implantation. Thus, the collapse resistance of the NGCs scaffold is crucial to provide a stable environment for the nerve repair and regeneration.^[^ ^5a^ ^]^ Figure 1f demonstrates that the SD cannot be compressed by 100 g of weight and maintained structural integrity. Moreover, the relatively thin wall of SDC can be easily collapsed to lose its shape and compressed to form a thin sheet that cannot recover to its original shape. In contrast, after being compressed by 100 g of weight, the SDCHC was completely closed and recovered after the weight was removed. Moreover, the mechanical durability of SDCHC was tested by successive compression cycles. The SDCHC exhibited high levels of compressive stress and can retain ≈85% of its initial compressive stress and good structural integrity even after undergoing 100 cycles of compression at 30% deformation (Figure 1g). Compared to the structural destruction of the SD and SDC, the SDCHC is easily restored to its original shape after the removal of external forces (Movie S1, Supporting Information). Furthermore, these findings revealed that the biomimetic versatile SDCHC exhibits high electrical conductivity, anisotropic structure, permeability, hydrophilicity, and collapse resistance.

### 2.3 SDCHC Facilitate Cell Outgrowth and Neurite Orientation In Vitro

**Figure 2:**
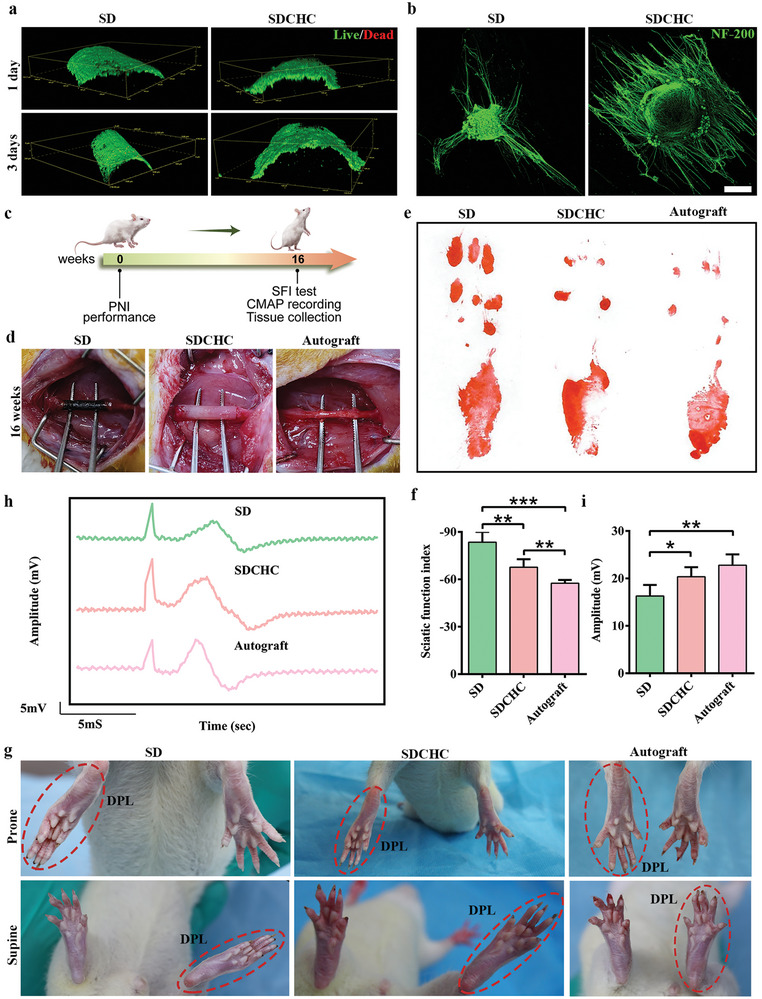
Bioactivity of each scaffold and functional recovery of transected sciatic nerves. a) Live‐dead staining of PC12 was cultured for 1 and 3 days on the surface of SD and SDCHC. Green represented live cells; red represented dead cells. b) Immunofluorescent staining of DRGs cultured for 10 days on the surface of SD and SDCHC. Scale bars: 50 µm. Green represented NF‐200. c) Schematic diagram of the animal experiments to test the therapeutic effect of SDCHC in rat models of PNI. d) Postoperative diagram of SD group, SDCHC group, and autograft group in rat sciatic nerve defects at 16 weeks after surgery. e) Digital camera images of the footprint in the SD group, SDCHC group, and autograft group at 16 weeks after surgery. f) Quantitative analysis of the SFI in the SD group, SDCHC group, and autograft group (Data were presented as mean ± S.D. *p < 0.05; **p < 0.01; ***p < 0.001. *p‐value* was generated by ANOVA and Tukey's test. *n* = 5 for SD group; *n* = 5 for SDCHC group; *n* = 5 for autograft group). g) Digital camera images of toe spread in the SD group, SDCHC group, and autograft group at 16 weeks after surgery. DPL: damage to the posterior limb. h) Electrophysiological recordings of CMAP for various implants in the SD group, SDCHC group, and autograft group at 16 weeks after surgery. i) Quantitative analysis of amplitude at CMAP in the SD group, SDCHC group, and autograft group (Data were presented as mean ± S.D. *p < 0.05; **p < 0.01; ***p < 0.001. *p‐value* was generated by ANOVA and Tukey's test. *n* = 5 for SD group; *n* = 5 for SDCHC group; *n* = 5 for autograft group).

We selected PC12 cells as model cells to investigate the biological performance of each scaffold. PC12 cells were cultured on the surface of SD and SDCHC to study their growth using live‐dead staining and were then observed in 3D view by confocal laser scanning microscopy (CLSM). **Figure** **2**a illustrates that the CLSM images revealed that PC12 cells seeded on the surface of the SD and SDCHC showed strong green fluorescence and weak red fluorescence at 1 and 3 days. This phenomenon indicates that SD and SDCHC exhibit good biocompatibility.

The dorsal root ganglion (DRG) as a model of primary neurons has been widely studied in many central or peripheral nerve research studies. Therefore, the DRGs were isolated and grown on the surfaces of SD and SDCHC to investigate the effects of anisotropic surfaces. After culturing for 10 days, DRGs were examined using immunofluorescence staining with neuronal‐specific markers of NF‐200. Figure 2b illustrates that the neurites of DRGs were randomly distributed and few in number on the surface of SD. Conversely, the neurites of the DRGs grew well parallel to each other under the guidance of the aligned microchannels on the surface of the SDCHC. These findings suggested that the SDCHC, with its parallelism of microchannels, aided in determining the course of neurite extension.

### 2.4 Repair Effects of SDCHC in the Model of Peripheral Nerve Injury

Individual NGCs (SD and SDCHC) of 12 mm length were prepared and implanted into the 10 mm nerve defect sites of SD rats to test the promoting effect of anisotropic electroactive SDCHC on peripheral nerve regeneration. The autograft will be used as a positive control by rotating the severed nerve by 180° and reimplanting it into the defect area. We looked at sciatic nerve regeneration in the SD, SDCHC, and autograft groups at 16 weeks after transplantation (Figure 2c). Figure 2d demonstrates that NGCs were still present at the site of the nerve defect in all rats without apparent septic accumulation or inflammation at 16 weeks. The hematoxylin‐eosin (HE) image shows a cross‐section of regenerating nerves in the central position to confirm further the absence of significant inflammatory cell infiltration (with large nucleus and deep staining) in all groups (Figure S7, Supporting Information). These results indicated that it had good compatibility and minimized immune responses as a platform for nerve regeneration.

Then, walking track analysis is critical in assessing motor function recovery after NGC implantation. The study's goal was to evaluate the sciatic function index (SFI), which was calculated using the footprint parameters.^[^ ^8d^ ^]^ Generally, the SFI value reflects the degree of peripheral nerve dysfunction, varying from −100 to 0. An SFI of −100 represents a complete loss of function, and an SFI of 0 represents a normal function. At 16 weeks postoperatively, the footprints of the rats in different groups could be clearly observed (Figure 2e). Statistical analysis showed that the SDCHC group had a significantly improved SFI compared to the SD group, whose SFI was more similar to the autograft group (Figure 2f). Further behavioral visual observation of toe spread revealed that functional recovery was significantly greater in the autograft and SDCHC groups than that in the SD group (Figure 2g).

Electrophysiological measurements were performed to measure the electrical transduction of regenerated nerves by monitoring the compound muscle action potential (CMAP) to assess the functional recovery of sciatic nerve defects with different groups.^[^ ^5a^ ^]^ The representative curves of CMAP are presented in Figure 2h. Statistical analysis demonstrated that the SDCHC group exhibited a similar amplitude and latency value to that of the autograft group, while a higher amplitude and a shorter latency than those in the SD group (Figure 2i; Figure S8, Supporting Information). These results show that SDCHC scaffold exhibited the potential to promote the recovery of electrophysiological function, which can be comparable with the performance of the autograft group.

**Figure 3:**
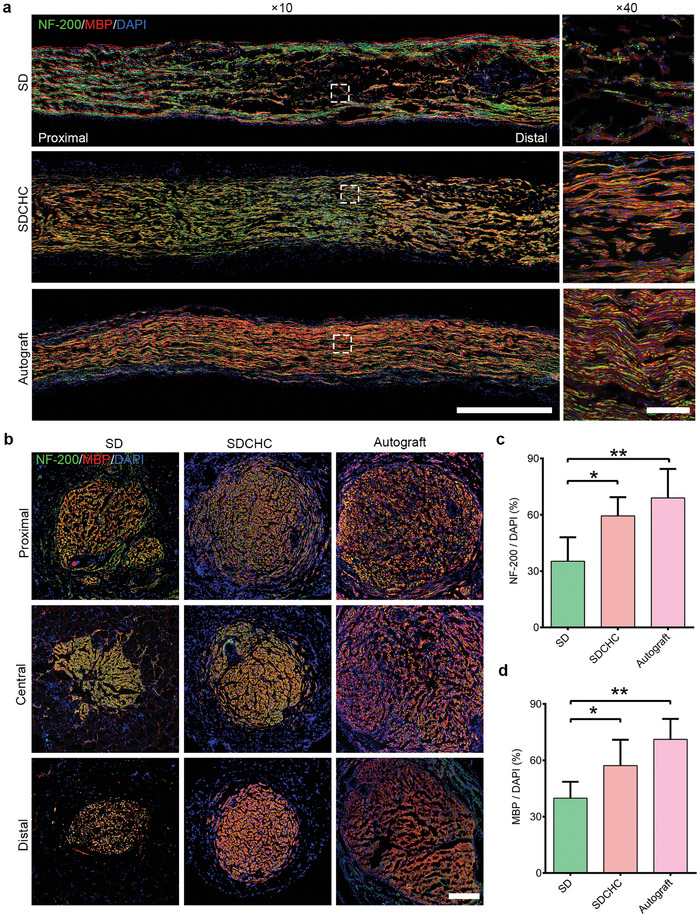
Histological changes in injured sciatic nerve at 16 weeks after surgery. a) Immunofluorescence staining of longitudinal sections of regenerating nerves in the SD, SDCHC, and autograft groups. Scale bars: 1000 µm at ×10 magnification; 100 µm at × 40 magnification. Green represents nerve fiber; red represents myelin sheath; blue represents cell nucleus. b) Immunostaining images of transverse sections of nerve fibers and myelin sheath in the SD, SDCHC, and autograft groups at 16 weeks after surgery. Scale bars: 200 µm. Green represents nerve fiber; red represents myelin sheath; blue represents cell nucleus. Statistical analysis of c) NF‐200‐positive area/DAPI area and d) MBP‐positive area/DAPI area at the distal of SD, SDCHC, and autograft groups. (Data were presented as mean ± S.D. *p < 0.05; **p < 0.01; ***p < 0.001. *p‐value* was generated by ANOVA and Tukey's test. *n* = 5 for SD group; *n* = 5 for SDCHC group; *n* = 5 for autograft group).

Immunofluorescence staining was performed on the longitudinal and transverse sections in the central region of the isolated nerve tissue to investigate further the effect of the SDCHC on nerve regeneration at the cellular level.^[^ ^26^ ^]^ In the image, green represents NF‐200 staining specific for neurofilament, red represents MBP staining specific for Schwann cells, and blue represents DAPI staining for nuclei. **Figure** **3**a demonstrates that NF‐200 and MBP proteins were expressed from the proximal stump to the distal stump in all groups, confirming the ingrowth of nascent axons and the form of myelin sheath. The distribution of green and red fluorescence in the SDCHC group was significantly greater than that in the SD group and similar to that in the autograft group. Additionally, the fluorescence structure in the SD group was disordered and irregular compared with the orderly structure of regenerated axons and myelin sheath in the SDCHC group and the autograft group. Moreover, it is revealed that the staining fluorescence intensities of NF‐200 and MBP in the SDCHC group at 16 weeks after surgery were high from proximal to distal regions and appeared almost similar to those in the autograft group. The staining intensities of NF‐200 and MBP were obviously reduced in the distal areas of the SD group (Figure 3b–d; Figure S9, Supporting Information). This result further suggests that the anisotropy and conductive microenvironment provided by biomimetic anisotropic and electroactive SDCHC may play a certain role in internal guidance pathways and promoting nerve regeneration.

**Figure 4:**
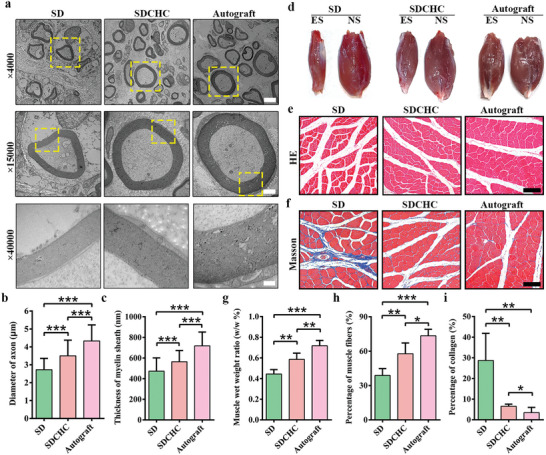
Histologic analyses of sciatic nerve and atrophy of GM muscle in the SD, SDCHC, and autograft groups at 16 weeks after surgery. a) TEM images of the myelin sheath in the central segment of the regenerated nerve. Scale bars: 20 µm at × 4000 magnification; 1 µm at × 15 000 magnification; 0.25 µm at × 40 000 magnification. d) Images of GM muscle of experimental side (ES) and normal side (NS). e) HE images in the central segment of the GM muscle. Scale bars: 100 µm. Red represents muscle fiber. f) Masson's images in the central segment of GM muscle. Scale bars: 100 µm. Red represents muscle fiber; blue represents collagen fiber. Statistical analysis of b) regenerated axon diameter, c) myelin sheath thickness, g) muscle wet weight ratio, h) the percentage of gastrocnemius muscle fibers, and i) the percentage of collagen fibers. (Data were presented as mean ± S.D. *p < 0.05; **p < 0.01; ***p < 0.001. *p‐value* was generated by ANOVA and Tukey's test. *n* = 5 for SD group; *n* = 5 for SDCHC group; *n* = 5 for autograft group).

Transmission electron microscopy (TEM) was further applied to observe the detailed structure of the regenerating nerves.^[^ ^5a^ ^]^ In high magnification, TEM images of subcircular or irregular oval structures, which are the myelin structures of regenerating nerves, can be observed. The structure of the wrapping of the myelin sheath is the axons of the regenerating nerves. **Figure** **4**a depicts that all groups exhibited the presence of myelin sheaths at 16 weeks postoperatively. Notably, regenerated axons within the SDCHC and autograft groups were predominantly surrounded by clear, thick, and electron‐dense myelin sheaths. In contrast, the SD group demonstrated thin and loosely arranged myelin sheaths. Statistically, the axon diameters in the SDCHC groups were significantly larger than those in the SD group and thinner than those in the autograft groups (Figure 4b). Furthermore, the myelin sheath thickness in the SDCHP groups was much thicker than that in the SD group and thinner than that in the autograft groups (Figure 4c).

After a sciatic nerve injury, atrophy of the gastrocnemius (GM) muscle occurs due to the loss of innervation and trophic effects accompanied by the deposition of large amounts of collagen.^[^ ^26^ ^]^ The regeneration degree of the sciatic nerve is reflected and analyzed by the muscle weight ratio, HE staining, and Masson's trichrome staining in the histological structural changes of the GM muscle. Figure 4d confirms that the GM muscles isolated from the experimental side (ES) showed varying degrees of atrophy compared to the contralateral normal side (NS). Particularly, those in the SD group were much lighter than those in the other groups. Moreover, the muscle fibers of the cross‐sectional areas in the SD group demonstrated that prominent muscle atrophy and reduction were observed by HE staining (Figure 4e). However, no apparent degradation of the GM muscle was detected in either the SDCHC group or the autograft group. Furthermore, widespread collagen deposition was noticed around the atrophied muscle fibers in the SD group, as determined by Masson's trichrome staining (Figure 4f). Conversely, the SDCHC group exhibited a decrease in collagen percentage, similar to the autograft group. According to quantitative analysis, in the SDCHC group, the wet weight ratio of GM muscle recovered to ≈59% ± 0.061%, which was much higher than that in the SD group (Figure 4g). Statistically, the SDCHC group had a significantly higher percentage of muscle fibers and a considerably lower percentage of collagen than the SD group (Figure 4h,i). Overall, these findings indicated that the biomimetic anisotropic and electroactive SDCHC with both topographical and electrophysiological cues exhibited comparable nerve regeneration performance to the autograft group. The biomimetic anisotropic and electroactive SDCHC with aligned fibers microstructures well matched with the structure and physicochemical properties of native nerve tissue and provided suitable support and guideness for cell growth, functionality, and maturation in nerve repair. Thus, the SDCHC scaffold has greater superiority for promoting nerve regeneration, facilitating the transmission of electrical signals, slowing the atrophy of GM muscle, and even facilitating functional recovery. Further research is needed to exploit the underlying mechanism.

### 2.5 Design and Fabrication of Biomimetic Anisotropic and Electroactive SDCHP

**Figure 5:**
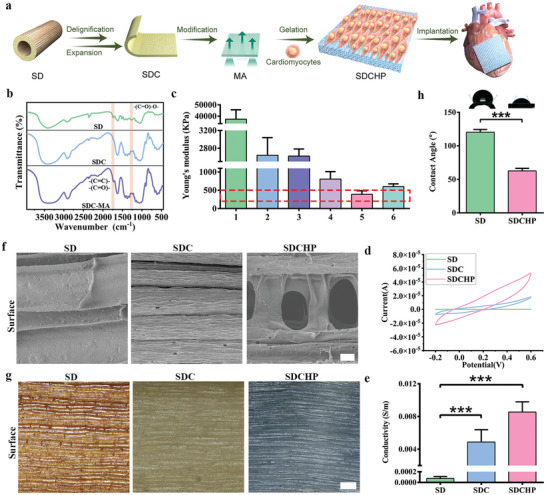
Fabrication and characterizations of the SDCHP. a) Schematic of the construction and application of the SDCHP for MI. b) FT‐IR spectra of the SD, SDC, and SDC‐MA. c) Analysis of the mechanical of the SD, SDC, and SDCHP. The red box represents the natural cardiac tissue modulus. Column 1: SD. Column 2: SDC. Column 3: SDC‐MA. Column 4: SDCHP with 15% PAA. Column 5: SDCHP with 15% PAA/5% GelMA. Column 6: SDCHP with 15% PAA/10% GelMA. h) Analysis of contact angle of the SD and SDCHP (data are presented as mean ± S.D; *p < 0.05, **p < 0.01, ***p < 0.001; unpaired *t*‐tests were used for comparisons; *n* = 3 independent samples). d) CV curves of the SD, SDC, and SDCHP at 100 mV s^−1^ scan rates. e) Analysis of electrical conductivity of the SD, SDC, and SDCHP. (Data were presented as mean ± S.D. *p < 0.05; **p < 0.01; ***p < 0.001. *p‐value* was generated by ANOVA and Tukey's test, *n* = 5 independent samples). f) SEM image on the surface of the SD, SDC, and SDCHP. Scale bars: 10 µm. g) Digital microscope images on the surface of the SD, SDC, and SDCHP. Scale bars: 100 µm.

We extend SD's application scenarios for another typical soft excitable tissue‐cardiac tissue engineering to further demonstrate its designability and versatility. During the preparation process, we discovered that the SD had good biocompatibility and that the SDC from SD had an anisotropic structure, which closely resembled that of myocardial tissue, making it promising for functional ECPs. However, the SD is an originally stiff and nonconductive natural biomaterial that does not meet the biomechanical and electrical conductivity requirements of natural myocardium. Moreover, the SD has a limited contact area with the heart, which is not conducive to providing mechanical support for repairing and regenerating myocardial tissue. Therefore, making an irreconcilable trade‐off between microstructural anisotropy, electroactivity, and biocompatibility is challenging. Thus, we suggest that the SDC from the SD should be expanded into a lamellar structure to increase the contact area with the heart and bridge healthy myocardium across the scar region (**Figure** **5**a). After expanding the SDC, we modified it with methacrylic anhydride (MA) to provide the SDC with more chemical functional groups.^[^ ^12^, ^27^ ^]^ In Figure 5b, FT‐IR analysis shows that the peaks at 1735 cm^−1^ of SDC almost disappeared with a significant decrease in peak intensities compared to those of SD, corresponding to the carboxyl groups of hemicellulose. Meanwhile, these SDC‐MA peaks were assigned to MA functional groups (carboxyl and alkyne), and their characterizations confirmed the successful grafting of MA onto the SDC. Following that, we achieved the desired biomimetic anisotropy, electroactivity, and modulus of elasticity by in situ gelation of conductive acrylic acid and various concentrations of GelMA on the surface of the SDC‐MA composite. Eventually, biomimetic anisotropic electroactive hydrogels, such as the SDCHC for PNI and the SDCHP for MI, were developed to demonstrate the designability and versatility of the SD. This capability underscores SD's potential to enable a wide spectrum of bioapplication scenarios tailored for soft tissue repair.

### 2.6 Characterization of Biomimetic Anisotropic and Electroactive SDCHP

An ideal scaffold for ECP should have an appropriate modulus of elasticity similar to natural heart tissue and be capable of synchronous contraction of cardiomyocytes in vitro.^[^ ^28^ ^]^ Therefore, the mechanical properties of each scaffold were evaluated using the compression test and Young's modulus method. A series of soft SDCHPs were developed by in situ polymerization with acrylic acid and different concentrations of GelMA to mimic the natural myocardial microenvironment further. Figure 5c shows that Young's modulus of SD, SDC, SDC‐MA, SDCHP with 15% PAA, SDCHP with 15% PAA/5% GelMA, and SDCHP with 15% PAA/10% GelMA were 37 262 ± 8376, 2641 ± 399, 2622 ± 159, 807 ± 205, 389 ± 100, and 601 ± 74 KPa, respectively. These results revealed that such an appropriate Young's modulus of the SDCHP with 15% PAA/5% GelMA was well matched to the natural myocardium at the end of diastole, which has a modulus of 200 to 500 KPa during cardiac diastole.^[^ ^29^ ^]^ This SDCHP may provide the necessary biomechanical support for further direct application as ECP, which can have a significant impact on fundamental cell behavior such as cell proliferation, migration, maturation, and differentiation.^[^ ^30^ ^]^

According to tissue engineering approaches, preparing engineered biomaterials typically involves creating a scaffold that closely mimics the native tissue environment. Electrically excitable cells such as neurons and cardiomyocytes can rapidly depolarize away from the resting membrane potential under physiological conditions, where propagating the electrical signal is essential for the physiological function of the tissue.^[^ ^31^ ^]^ Accordingly, the SDCHC and SDCHP were designed to provide an electrical conduction pathway that allows for efficient cell‐cell electrical interaction following transplantation. The electrical conductivity of each scaffold was then assessed using CV (Figure 5d). Like the SDCHC, the electrical conductivity of SDCHP was found to be (8.5 ± 1.3) × 10^−3^ S m^−1^, which is similar to that of the natural myocardium (Figure 5e).^[^ ^32^ ^]^ We further investigated some related tests of mechanical properties and electrical conductivity across the vertical (R) and parallel (L) direction of samples to further verify the anisotropic properties (Figure S10, Supporting Information). In comparison to those in R‐direction, each sample exhibits higher mechanical properties and lower electrical conductivity in L‐direction in the SD, SDC, and SDCHP, suggesting the anisotropic mechanical and electrical properties of SD‐derived scaffolds with ordered parallel microstructures.

Oriented tissues (e.g., neurons, cardiomyocytes) are clearly aligned with the distinct spatial organization of their ECM and residing cells to achieve unique physiological functions.^[^ ^33^ ^]^ Accordingly, the designs of SDCHC and SDCHP aim to mimic the native tissue environment to induce cell alignment. This alignment ultimately results in forming an orderly organizational structure, which can have profound effects on various cellular functions or behaviors.^[^ ^34^ ^]^ Figure 5f shows the SEM images of anisotropic structure and microporous structures on the SDCHP surface. The digital microscope detected the anisotropic structure on the wet surface of SDCHP, closely resembling that of myocardial tissue (Figure 5g).^[^ ^35^ ^]^ Additionally, the cross‐section SEM image revealed the presence of a 3D hierarchical microchannel structure, similar to that of SDCHC (Figure S11, Supporting Information). Such a biomimetic structure is proven to be crucial for endogenous cell infiltration and facilitating functional tissue regeneration in situ.^[^ ^34a^ ^]^

Next, we evaluated the hydrophilicity of each patch hydrogel material. Figure 5h reveals that the contact angle of SDCHP (63 ± 3.9°) exhibited improved hydrophilicity over the SD. Moreover, the SDCHP had a significant swelling rate, enabling it to absorb up to 80% of its initial weight (Figure S5b, Supporting Information). These results indicated that the biomimetic versatile SDCHP possesses appropriate elasticity, electrical conductivity, anisotropic structure, permeability, and hydrophilicity, which are critical for an effective cardiac patch.

### 2.7 SDCHP Facilitate CMs Survival, Orientation, and Functionalization In Vitro

**Figure 6:**
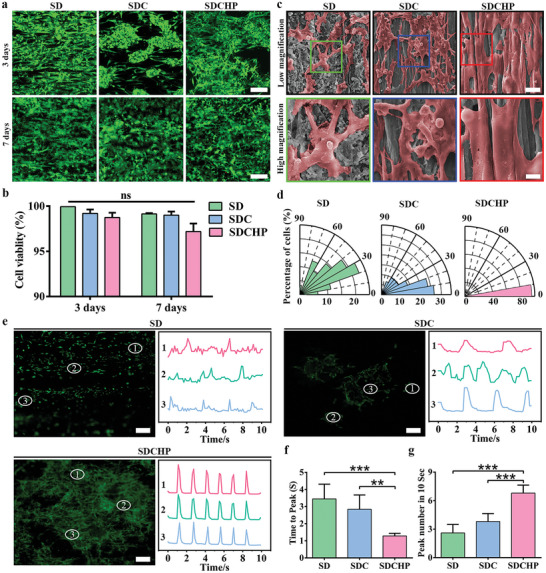
The morphological and functional characteristics of CMs on the surface of SD, SDC, and SDCHP. a) Live‐dead staining of CMs cultured for 3 and 7 days. Scale bars: 200 µm. Green represented live cells; red represented dead cells. b) Quantitative analysis of cell viability of CMs cultured for 3 and 7 days. Data are presented as mean ± S.D. statistical analysis (ns = no significance; *p*‐value was generated by ANOVA and Tukey's test; *n* = 5 independent samples). c) SEM images show the morphology of CMs cultured for 7 days. Scale bars: 20 µm. Red represented CMs. d) Statistical analysis of morphological elongation of CMs cultured for 7 days. e) Ca^2+^ transient analysis of CMs cultured for 7 days. Green represented CMs. The Ca^2+^ transient propagation f) time to peak and g) peak numbers of CMs cultured for 7 days. (Data were presented as mean ± S.D. *p < 0.05; **p < 0.01; ***p < 0.001. *p‐value* was generated by ANOVA and Tukey's test, *n* = 5 independent samples).

The cytotoxicity was tested by live‐dead staining to evaluate the biocompatibility of the hydrogel. **Figure** **6**a represents the live‐dead staining images, showing that cardiomyocytes (CMs) survived (green), with few dead cells (red) at 3 and 7 days on each scaffold (SD, SDC, and SDCHP). The cell viability of the CMs was comparable across scaffolds, and the overall percentage of living cells was greater than 90% after 7 days of culture (Figure 6b).

Constructing topographical cues that mimic the anisotropic structure of natural cardiac tissues is crucial for inducing the orientation of CMs in tissue engineering.^[^ ^36^ ^]^ After 7 days of culture, the CM morphology was observed and evaluated using SEM. In Figure 6c, the CMs were randomly distributed on the SD surface without apparent directionality guidance. Conversely, the CMs exhibited an elongated shape with the anisotropic microstructure on the SDCHP surface. The statistical results implied that the high percentage of CMs on the SDCHP surface could be aligned and oriented in the same direction along aligned microchannels, closely resembling the organizational structure of natural cardiac tissue (Figure 6d).

We examined the changes in calcium signaling after 7 days of culture to investigate the effect of the conductive microenvironment on the systolic synchronization of CMs further.^[^ ^37^ ^]^ Calcium signaling changes of CMs were recorded using Fluo‐4 AM as a calcium indicator and randomly selecting three different points to measure the calcium transients on each scaffold. In Figure 6e and Movie S2 (Supporting Information), weak and disorderly transient signal changes between CMs on the surface of SD and SDC were observed. In contrast, faster and more regular transient signal changes between CMs on the surface of SDCHP were detected. Compared to the other groups, the transient calcium signal changes of the CMs on the surface of SDCHP had a statistically significantly lower time to peak and a considerably higher number of peaks in 10 s (Figure 6f,g). These results implied that the SDCHP would induce the oriented and accelerated functionalization of the CMs, providing a supportive environment for cell growth without significant adverse reactions when used in the field of tissue engineering.

### 2.8 Repair Effects of SDCHP ECP in Rat MI Models

**Figure 7:**
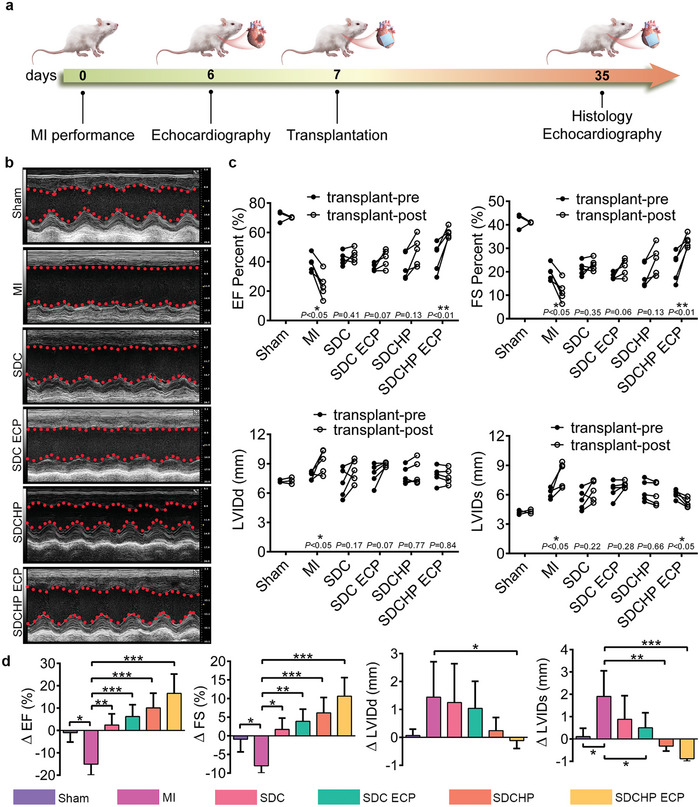
Echocardiographic evaluation of a cardiac function at pre‐transplantation and 4 weeks post‐transplantation in the sham, MI, SDC, SDC ECP, SDCHP, and SDCHP ECP groups. a) Schematic diagram of the animal experiments to test the therapeutic effect of SDCHP ECP in rat models of MI. b) Representative echocardiography images of the left ventricle at 4 weeks post‐transplantation. c) Changes of FS, EF, LVIDs, and LVIDd before transplantation and 4 weeks after transplantation. (Data were presented as mean ± S.D. *p < 0.05; **p < 0.01; ***p < 0.001. *p‐value* was generated by ANOVA and Tukey's test. *n* = 3 for sham group; *n* = 5 for MI group; *n* = 5 for SDC group; *n* = 5 for SDC ECP group; *n* = 5 for SDCHP group; *n* = 5 for SDCHP ECP group). d) Statistical analysis of the changes of FS, EF, LVIDs, and LVIDd during the 4‐week transplantation period. (Data were presented as mean ± S.D. *p < 0.05; **p < 0.01; ***p < 0.001. *p‐value* was generated by ANOVA and Tukey's test. *n* = 3 for sham group; *n* = 5 for MI group; *n* = 5 for SDC group; *n* = 5 for SDC ECP group; *n* = 5 for SDCHP group; *n* = 5 for SDCHP ECP group).

The model of MI was established in Sprague–Dawley (SD) rats by left anterior descending artery (LAD) ligation according to our previous method to investigate the therapeutic performance of SD‐derived ECPs transplantation in vivo. In our study, the SD rats were treated in the following groups: pure scaffold transplantation (SDC and SDCHP) and ECP transplantation (SDC ECP and SDCHP ECP). At 1 week postoperatively, MI rats with less than 30% left ventricular fractional short (LVFS) value were selected and ECPs were transplanted into a rat MI model (Figure S12, Supporting Information). The effects of the different treatments were evaluated after 4 weeks of transplantation using histology and M‐mode echocardiographic measurements (**Figure** **7**a). As shown in Figure 7b, the images of the echocardiographic and the echocardiographic video reveal that bare contraction activity occurred in the MI and SDC groups. Conversely, stronger contractile activity of the left ventricular anterior wall appeared in the SDCHP and SDCHP ECP groups. Next, M‐mode echocardiographic data were further statistically analyzed to assess cardiac function, including LVFS, left ventricular ejection fraction (LVEF), left ventricular internal diameter at end‐systole (LVIDs), and left ventricular internal diameter at end‐diastole (LVIDd). Figure 7c,d shows the apparent deterioration in cardiac function and the increase in left ventricular dilatation observed in the MI group. With ECP transplantation, ΔFS % and ΔEF % increased slightly in the SDC and SDC ECP groups, while they were highest in the SDCHP ECP group. Consistent with the promotion in EF and FS, LVIDd and LVIDs are efficiently prevented on negative LV dilation in SDC and SDCHP ECP group. The above results indicate that SDCHP ECP with biomimetic microstructure and mechanoelectrical cues exhibits superior potential for restoring cardiac function after MI.

**Figure 8:**
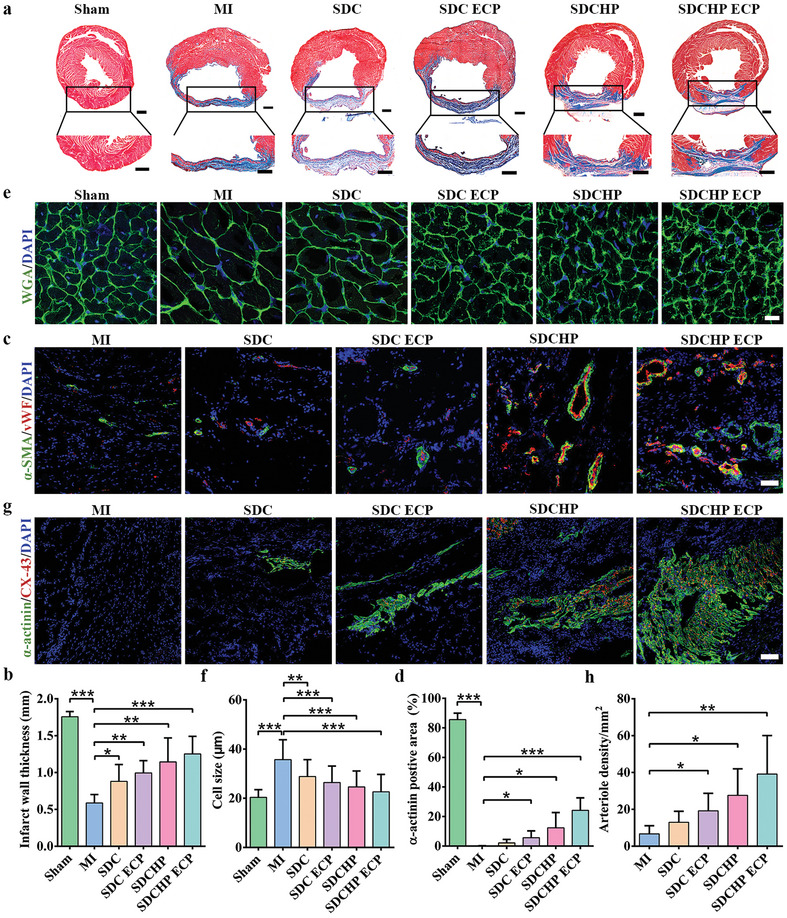
Histological evaluations for cardiac sections after 4 weeks post‐transplantation in the sham, MI, SDC, SDC ECP, SDCHP, and SDCHP ECP groups. a) Masson's trichrome staining for cardiac sections. Scale bars: 1000 µm. Red represented cardiomyocyte, and blue represented collagen fiber. e) Representative histological immunofluorescent staining of wheat germ agglutinin (WGA) showed cardiomyocyte boundaries in the border area. Scale bar: 20 µm. Green (WGA) represented cardiomyocyte boundaries. g) Immunofluorescent staining of CX‐43 and α‐actinin proteins in the infarct areas. Scale bars: 100 µm. Green represented α‐actinin; red represented CX‐43. c) Representative histological immunofluorescence staining of vWF and α‐SMA proteins in infarct areas. Green represented α‐SMA; red represented vWF. Scale bars: 50 µm. Quantitative analysis of b) wall thickness of left ventricular anterior wall, f) cardiomyocyte size, h) arteriole density, and d) α‐actinin positive area based on the immunostaining images. (Data were presented as mean ± S.D. *p < 0.05; **p < 0.01; ***p < 0.001. *p‐value* was generated by ANOVA and Tukey's test. *n* = 3 for sham group; *n* = 5 for MI group; *n* = 5 for SDC group; *n* = 5 for SDC ECP group; *n* = 5 for SDCHP group; *n* = 5 for SDCHP ECP group).

Following MI, the ischemic environment created by coronary artery occlusion can result in irreversible cardiomyocyte death and fibrous tissue formation in the infarcted area.^[^ ^38^ ^]^ Cardiac morphological changes were assessed using Masson's trichrome staining to determine the infarct area and infarct wall thickness in the infarct area at 4 weeks. In **Figure** **8**a, the infarct region of the MI group contained a significant amount of fibrous tissue (blue) and bare myocardial tissue (red), with the MI group showing the most severe fibrosis. No significant reduction in infarct region was observed in the SDC, SDC ECP, and SDCHP groups. By contrast, transplantation with the SDCHP ECP resulted in a significant decrease in infarct area and an increase in infarct wall thickness compared to the MI group, indicating superior performance of MI repair with SDCHC ECP over SDC and SDC ECP alone (Figure 8b; Figure S13a, Supporting Information).

Restoring the blood supply, which is critical for tissue repair, can provide the blood flow to deliver sufficient nutrients and oxygen to the infarcted area of the heart, allowing the remaining myocardial tissue to be salvaged and the rat's cardiac function to be restored.^[^ ^39^ ^]^ Revascularization was assessed using vWF‐positive microvessels and vWF/α‐SMA‐positive arterioles to evaluate the angiogenesis in the infarct area. Figure 8c presents the immunofluorescence staining, which reveals that arterioles with complete and clear structures had significant microvascular regeneration in different groups. The SDC ECP, SDCHP, and SDCHP ECP groups exhibited a substantially higher density of revascularization compared to the MI groups. According to the statistical analysis, the number of blood vessels in the SDCHP ECP group was approximately sixfold higher than that in the MI group, indicating that it can promote the formation of microvessels (Figure 8d). Conductive scaffolds are recognized for their potential to restore the conductive microenvironment and promote neovascularization in the infarcted area. Arteriole density was higher in the SDCHP and SDCHP ECP groups, implying that the desired conductive microenvironment could induce vascularization in the infarcted area.

Cardiac compensates for hypertrophy to maintain normal heart function after MI, increasing cardiac stress and leading to ventricular remodeling. To further assess the state of cardiac hypertrophy, the inhibition of cardiac hypertrophy in the different groups was compared using wheat germ agglutinin (WGA) staining. In Figure 8e, the cross‐sectional area of the cardiac was significantly increased in the MI group compared to that in the sham group, indicating critical ventricular hypertrophy. Four weeks after the transplantation, the state of cardiac hypertrophy was inhibited in pure scaffold transplantation and ECP transplantation groups compared to that in the MI group. According to the statistical analysis, each transplant group was effective in inhibiting cardiac hypertrophy and restoring heart function, which is consistent with echocardiographic data (Figure 8f).

To further evaluate the remaining myocardial cells in the infarct area, anti‐CX‐43 antibody and anti‐α‐actinin antibody were used to label the electron coupling protein and the cardiac‐specific protein, respectively, after 4 weeks of transplantation.^[^ ^40^ ^]^ In Figure 8g, the CX‐43‐positive and the α‐actinin‐positive areas in the infarct region of the MI group contained bare residual myocardial cells after surgery, but they were significantly upregulated in the other transplant groups. Notably, the area of CX‐43 and α‐actinin expression in the infarct area increased in the SDCHP ECP group, indicating a higher amount of residual myocardial and electrical integration (Figure 8h; Figure S13b, Supporting Information). These outcomes suggest that the SDCHP ECP significantly promoted vascular regeneration, improved residual myocardium, attenuated cardiac hypertrophy, and improved cardiac function after MI.

## 3 Conclusion

In this work, inspired by the naturally anisotropic structure of the nerve tract and myocardial fiber bundle, we successfully tailored the originally stiff, hydrophobic, and nonconductive SD into biomimetic versatile anisotropic electroactive hydrogels for soft excitable tissue repair. Moreover, to address the unique needs of repairing damaged tissue, we employed a robust yet simple top‐down approach to customize the SDCHC to bridge the sciatic nerve gap for PNI and the SDCHP to bridge healthy myocardium across the scar region for MI. Consequently, the biomimetic versatile hydrogel exhibited excellent properties, including well biocompatibility, highly anisotropic structure, excellent conductivity, mechanical durability, and permeability. Evidence from in vitro experiments indicates that these anisotropic hydrogels govern the orientation of cells and induce cell elongation. This phenomenon was attributed to the SDC preserving the hierarchical microchannel structure, which allows the hydrogel to guide cells for directional migration and spatial organization. In vivo, animal experiments demonstrated that SDCHC performed perfectly in significantly accelerating nerve regeneration, restoring electrical signaling, slowing GM muscle atrophy, and promoting motor function recovery in the PNI rats. Similarly, the SDCHP greatly facilitated the angiogenesis, inhibited the fibrosis, prevented the hypertrophy, and improved the cardiac function in the MI rats. In the future, fern‐derived scaffold materials could have the potential to meet large‐scale requirements for not only soft tissues but also hard tissues (e.g., bone and teeth). We anticipate that extensive future work will be undertaken to build a biomimetic versatile biological platform on the existing knowledge and to advance the field toward sustainable development.

## 4 Experimental Section

### Materials

*Dicranopteris linearis* was collected from the mountains of southern China. Sodium hypochlorite, vinyltrichlorosilane (VTC), methacrylic anhydride (MA), acrylic acid (AA), N, N’‐methylenediacrylamide (MBA), ammonium persulfate (APS), were purchased from Macklin (China). N, N, N’, N’‐tetramethylethylenediamine (TEMED) was obtained from Beijing Leagene Biotechnology Co., Ltd. Lignin, hemicellulose, and cellulose content assay kit were purchased from BOXBIO (China). Neuronal base medium, B‐27, L‐Glutamine, Dulbecco's modified Eagle's medium (DMEM)/high‐glucose medium, Certified gold fetal bovine serum (3021A, Umedium), and trypsin were purchased from Gibco (USA). The primary antibodies against α‐actinin (ab9465), connexin 43 (CX‐43, ab11370), NF‐200 (ab207176), MBP (NE1019), and von Willebrand factor (vWF, ab6994) were obtained from Abcam (UK). The primary antibody against alpha‐smooth muscle actin (a‐SMA, BM0002) was ordered from Boster Biological Technology (China). Alexa Fluor‐568 donkey anti‐rabbit IgG (H&L) and Alexa Fluor‐488 donkey anti‐mouse IgG (H&L) were from Thermo Fisher (USA). Murine pheochromocytoma (PC12) neuronal cells were purchased from Jennio biological technology (Guangzhou, China). Scheme 1 was created with Figdraw.

### Animal Sources

Sprague‐Dawley (SD) rats (male, weight 250 ± 30 g, 8–10 weeks) were provided from the Laboratory Animal Center of the Academy of Southern Medical University (China) under the ethics committee guidelines, and laboratory animals were approved by the Southern Medical University Animal Ethics Committee (SYXK (yue)2021‐0167).

### Synthesis of GelMA

Gelatin was completely dissolved in PBS at 50 °C. Methacrylic anhydride was added dropwise into the gelatin solution while stirring vigorously. The mixture was further stirred at 40 °C for 3 h and the reaction was terminated by dilution with PBS. The solution was subjected to dialysis with a 12–14 kDa membrane (USA) at 40 °C for 5 days. After dialysis, the solution was lyophilized and stored at ‐20 °C.

### Lignin Removal from SD

Different types of delignification methods can be used. Herein, a section of SD was immersed in sodium hypochlorite to remove the lignin for several days. The sodium hypochlorite was changed once a day until the color of SD turned from brown to white and the SDC was prepared. Then, the SDC was rinsed 5 times with deionized water to remove any residual chemicals, and placed in deionized water for backup.

### Fabrication of SDCHC

SDC was modified by immersion in VTC overnight, followed by cleaning 5 times with deionized water. Then add AA, MBA, APS, TEMED, and deionized water at 70 °C for 30 min. After the cross‐linker was finished, it was placed in PBS solution and the excess PAA was removed.

### Fabrication of SDCHP

SDC was flattened with two pieces of filter paper and then modified by immersion in 1% MA overnight, followed by cleaning 5 times with deionized water. Then add AA, GelMA, MBA, APS, TEMED, and deionized water at 70 °C for 30 min. After the cross‐linker was finished, it was placed in PBS solution and the excess PAA was removed.

### Material Characterization

An FT‐IR spectrometer (Nicolet is10, Thermo Scientific, USA) was used to record FT‐IR spectra at wavelengths 500 and 4000 cm^−1^. SEM (S‐3000N, Hitachi, Japan) and digital microscope (DSX1000, Olympus, Japan) were used to observe the surface microstructure of different hydrogels. TEM (H‐7500, Hitachi, Japan) was used to observe the nerve myelin sheath. A universal materials testing system (34SC‐1, INSTRON, USA) was used to test the mechanical properties of different hydrogels and the cycling compressive tests with 30% deformation for 100 cycles. An electrochemical workstation (CS350 M, CorrTest Instruments, China) was used for electrical conductivity and electrochemical impedance spectroscopy (EIS) of different hydrogels. An optical contact angle meter (Theta Flex, Biolin Scientific, Finland) was used to measure the contact angle of different hydrogels. Scaffolds from different groups were soaked in PBS for 7200 min at room temperature until swelling equilibrium was reached. After the surface water had been wiped off, the scaffolds were weighed and the swelling ratio of the scaffold was determined according to the following equation. Swelling ratio: W % = (W_t_ − W_0_)/W_0_ ×100%. Here, W_t_, and W_0_ represent the weight of the scaffold at different time points after swelling in PBS and the weight of the dried scaffold respectively.

### Cell Culture

Primary cardiomyocytes (CMs) were isolated from the hearts of 2‐day‐old Sprague‐Dawley (SD) rats by the described method and cultured in medium (15% FBS, 1% penicillin‐streptomycin in DMEM). PC12 were cultured in growth medium (5% FBS, 15% HS, 1% penicillin‐streptomycin in DMEM/F12) using a six‐well cell culture plate (SORFA). Dorsal root ganglion (DRG) neurons were isolated from the lumbar spinal cord and left DRGs (L3‐6) of 1‐day‐old SD rats and cultured in medium (2% B‐27, 1% Gln, 1% penicillin‐streptomycin in neuronal base medium). The cells were placed in an incubator at 37 °C with 5% CO_2_ and the culture medium was changed once a day.

### Live‐Dead Staining

The collected CMs (5 × 10^5^ per cm^2^) and PC12 (5 × 10^5^ per cm^2^) were seeded on the surface of each scaffold for several days. Then the scaffolds were washed three times in PBS and incubated with live‐dead staining for 5 min. The scaffolds were kept moist and then photographed using confocal laser scanning microscopy (CLSM, Nikon, Japan).

### Immunofluorescence Staining of DRGs

DRGs were isolated from 1‐day‐old SD rats by the described method and cultured in a medium. The DRGs were seeded on the surface of each scaffold for 10 days. The DRGs were washed three times in PBS and fixed with 4% paraformaldehyde for 15 min. The samples were permeabilized with 0.2% Triton X‐100 for 30 min, followed by blocking with 2% BSA at room temperature for 60 min. After removal of the residual reagents, the samples were incubated with rabbit anti‐NF‐200 primary antibody (1:500) at 4 °C overnight, followed by anti‐rabbit secondary antibodies (1:500) for 2 h. The scaffolds were kept moist and then photographed using CLSM.

### The Morphology of CMs

The CMs were seeded on the surface of each scaffold for 7 days. After that, each scaffold was washed with PBS and then fixed in 2.5% glutaraldehyde for 7 days. The fixed cells were then gradient dehydrated with different concentrations of ethanol (50%, 60%, 70%, 80%, 90%, 95%, and 100%) and critical‐point drying. Finally, the morphology of CMs on each scaffold was photographed using SEM.

### Transient Calcium Analysis of CMs

The CMs were seeded on the surface of each scaffold for 7 days and stained with fluo4‐AM reagent (Life Technologies, USA) at 37 °C for 45 min to record calcium activity using a fluorescence microscope (Olympus BX53, Japan). The results of the video were analyzed using the Image J software.

### Implantation of ECPs into the Rat MI Model and Corresponding Evaluation

Adult male SD rats were anesthetized with isoflurane and then achieved thoracotomy and subjected to MI by ligating the proximal left anterior descending (LAD) artery as described method. The heart functions of rats were evaluated by echocardiography after 6 days of LAD ligation and then MI rats with less than 30% fractional short (FS) value were selected for the next transplantation experiments. Further, the CM‐seeded scaffolds (ECPs) were randomly implanted into the MI rats. MI rats were divided randomly into five groups (*n* = 5 rats per group), including the MI group, SDC group, SDC ECP group, SDCHP group, and SDCHP ECP group. Then the different ECPs were transplanted onto the epicardium in the infarction area. Guided by the anatomical understanding of the heart, anatomical landmarks, and visual cues were used to closely approximate the path of the myocardial fibers located below the left anterior descending artery (LAD) ligation point. The ECP was then fixed in place using 7‐0 sutures. The fiber orientation of the ECP was aligned as closely as possible to accord with the counterclockwise helical direction of the neighboring myocardial fibers. Besides, the sham group (*n* = 3 rats) was subjected to the same thoracotomy without LAD ligation.

### Cardiac Function Analysis

The function of the left heart in all groups of rats was assessed by Vevo2100 echocardiography with the software (Vevo2100, Visual Sonics). Four weeks after ECPs transplantation, the rats were anesthetized with isoflurane and M‐mode traces, and short‐axis views were recorded using an M250 transducer. Cardiac function parameters were derived from the average of three consecutive cardiac cycles measured by the device using M mode, including left ventricular ejection fraction (LVEF), left ventricular shortening fraction (LVFS), left ventricular end‐diastolic diameter (LVIDd), and left ventricular end‐systolic diameter (LVIDs). The formula was also shown as follows: LVEDV = [7/(2.4 + LVIDd)] × LVIDd^3, LVESV = [7/(2.4 + LVIDs)] x LVIDs^3, LVEF = 100 × (LVEDV – LVESV) / LVEDV.

### Histological Analysis of Cardiac Sections

All rats were sacrificed after 4 weeks of transplantation, and the hearts of different groups of rats were collected and cut into three cross‐sections of ≈0.4 mm from apex to atrium. The hearts were fixed in 4% paraformaldehyde overnight and dehydrated in 30% sucrose. Middle sections of the hearts were embedded in O.C.T and sectioned at 6 µm using a frozen slicer. Masson trichrome staining could stain the myocardium in red and collagen in blue to determine the histological features of myocardial infarction. Based on the Masson trichrome staining images, the infarct areas and the wall thickness of the infarct area were also measured and analyzed using Image J software.

### Immunofluorescence Staining of Cardiac Sections

The cardiac sections were washed three times in PBS to remove O.C.T, then permeabilized with 0.2% Triton X‐100 for 30 min and blocked with 2% Bovine Serum Albumin (BSA) at room temperature for 60 min. Subsequently, the primary antibodies of α‐actinin (1:500), CX43 (1:500), vWF (1:200), α‐SMA (1:200), and WGA (1:200) were used to cover cardiac sections at 4 °C overnight. All cardiac sections were incubated with the appropriate secondary antibody (1:500) for 2 h at room temperature. The nuclei were stained with DAPI (Sigma, USA). All tissues were observed and imaged using a fluorescence microscope (Olympus BX5, Japan).

### Implantation of NGC and Corresponding Evaluation

Adult male SD rats were used to establish models of peripheral nerve injury (PNI) and randomly divided into three groups: i) the SD group. ii) the SDCHC group. iii) the autograft group (*n* = 5 rats per group). All surgical procedures were performed under anesthesia using isoflurane. The left peripheral nerve was exposed by making an incision through the skin and muscle layers to remove it completely, leaving a gap of 10 mm. Next, to bridge the nerve defect, the proximal and distal of the defective peripheral nerve were implanted into a 12 mm NGC and tied to the epineuria sheath using 10‐0 nylon sutures under the microscope. In the autograft group, a 10 mm peripheral nerve gap was formed and then the transected nerve segment was re‐implanted and sutured. 4‐0 nylon sutures were used to suture the muscle and skin layers. At the chosen time post‐surgery, rats were observed and sacrificed.

### Walking Track Analysis

The walking track analysis was performed to assess the functional recovery in all groups at 16 weeks after surgery. The rats were guided to walk on a piece of paper and leave footprints after applying carbon ink to both hind feet. The parameters of the footprints were measured on both sides including the print length (EPL, NPL), toe spread (ETS, NTS), and intermediate toe spread (EIT, NIT). The SFI were calculated by a formula as follows: SFI = 38.3 [(EPL‐NPL)/NPL]+109.5 [(ETS‐NTS)/NTS]+13.3 [(EIT‐NIT)/NIT].

### Electrophysiological Assessment

The left peripheral nerve was exposed for testing of electrical conduction in the nerve. Electrical stimulation was applied to the proximal ends of the nerve segment, and CMAP was recorded at the gastrocnemius belly. Finally, the peak amplitude of CMAP and the latency of CMAP onset were measured and analyzed to be compared among groups.

### Morphometric Evaluation of Axonal Regeneration

All the middle portions of the regenerated nerves were retrieved at 16 weeks after surgery and fixed in a solution of 4 wt.% glutaraldehyde overnight. Transverse 50.0 nm thick ultrathin sections were obtained from nerve and stained with 5% uranyl acetate and 0.3% lead citrate. Next, all samples were examined by TEM (Hitachi‐7500, Japan) and measured and analyzed using Image J software.

### Analysis of Gastrocnemius Muscle Degeneration

The harvested gastrocnemius muscles (GM) were immediately weighed from the experimental sides (GM(E)) and normal side (GM(N)) in different groups at 16 weeks after surgery. The GM weight percentage was calculated by a formula as follows: GM weight (%) = GM (E)/GM (N) × 100%. The harvested gastrocnemius muscles of the left side were fixed in 4% paraformaldehyde overnight and dehydrated in 30% sucrose for 2 days. The gastrocnemius muscles were embedded in O.C.T and sectioned at 6 µm in the transverse direction using a frozen slicer. The gastrocnemius muscles were stained with hematoxylin and eosin (HE) and Masson trichrome staining. Based on HE and Masson trichrome staining images, the percentage of muscle fiber area and collagenous fiber were also observed under a microscope (BX53, Olympus, Japan). The percentage of muscle fiber (Pm) area was calculated using the following formula: Pm = Am/At× 100%, where Am was the area of muscle fibers in each field, and At was the total area of the image field. The percentage of collagenous fiber (Pc) area was calculated using the following formula: Pm = Ac/At× 100%, where Ac was the area of collagenous fibers in each field. At least five fields per section were randomly selected in the images and analyzed using Image J software.

### Immunofluorescence Staining of Axonal Regeneration Sections

Sixteen weeks after surgery, the axonal regeneration in NGC‐implanted rats was isolated, embedded in O.C.T, and sectioned at 6 µm in the transverse or longitudinal direction using a frozen slicer. The axonal sections were washed three times in PBS to remove O.C.T, then permeabilized with 0.2% Triton X‐100 for 60 min and blocked with 2% BSA at room temperature for 60 min. Subsequently, the primary antibodies of NF‐200 (1:200) and MBP (1:200) were used to cover axonal sections at 4 °C overnight, followed by incubation with the appropriate secondary antibody (1:500) for 2 h at room temperature. The nuclei were stained with DAPI. All tissues were observed and imaged using a fluorescence microscope. NF‐200‐positive and MBP‐positive areas were counted in the distal region in transverse sections using Image J (*n* = 5 rats per group). Additionally, the axonal sections were subjected to HE staining and imaged using a fluorescence microscope.

### Statistical Analysis

At least three independent replicates of different types of experiments were performed and statistically analyzed. The data were expressed as means ± standard deviations. GraphPad Prism 7.0 software and SPSS 20.0 software were used for statistical analysis. Unpaired *t*‐tests were used for comparisons between the two groups. Multiple comparisons were carried out using one‐way analysis of variance (ANOVA) and Tukey's post‐hoc analysis.

## Conflict of Interest

The authors declare no conflict of interest.

## Supporting information



Supporting Information

Supplemental Movie 1

Supplemental Movie 2

## Data Availability

Research data are not shared.
