# Supplementary material for: Biomimetic Versatile Anisotropic, Electroactive Cellulose Hydrogel Scaffolds Tailored from Fern Stem Serving as Nerve Conduit and Cardiac Patch
Source: Adv Sci (Weinh). 2024 Dec 4;12(4):2400002. doi: 10.1002/advs.202400002 (PMC11789595; doi:10.1002/advs.202400002)
Supplement: Supplementary file 1 — Supporting Information [file ADVS-12-2400002-s002.docx]

**Supporting Information**

**Biomimetic versatile anisotropic, electroactive cellulose hydrogel scaffolds tailored from fern stem serving as nerve conduit and cardiac patch**

*Qinghui Liang, Shuhui Chen, Shaofeng Hua, Weihong Jiang, Jiamian Zhan, Chunyi Pu, Rurong Lin, Yutong He, Honghao Hou*, Xiaozhong Qiu**

Guangdong Provincial Key Laboratory of Construction and Detection in Tissue Engineering, Department of Anatomy, School of Basic Medical Sciences, Southern Medical University, Guangzhou, Guangdong 510515, P. R. China.

* Address correspondence to:

Dr. Xiaozhong Qiu ([qqiuxzh@163.com](mailto:qqiuxzh@163.com)); Dr. Honghao Hou ([ss.hhh89@hotmail.com](mailto:ss.hhh89@hotmail.com))

**This Supporting Information file includes:**

Figure S1-S13


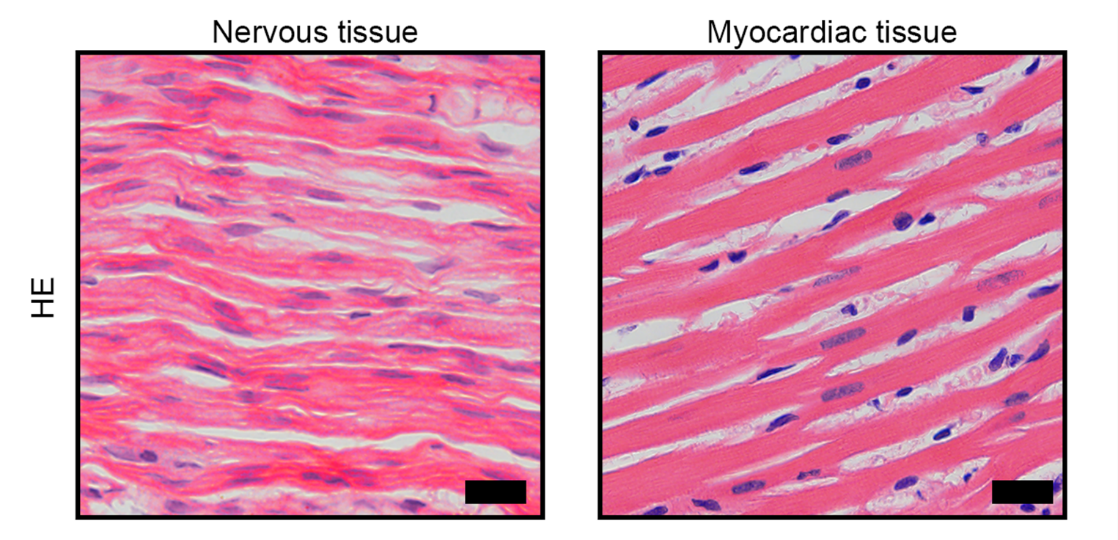


**Figure S1.** The HE image of natural nervous tissue and myocardial tissue. Scale bar: 20 μm.


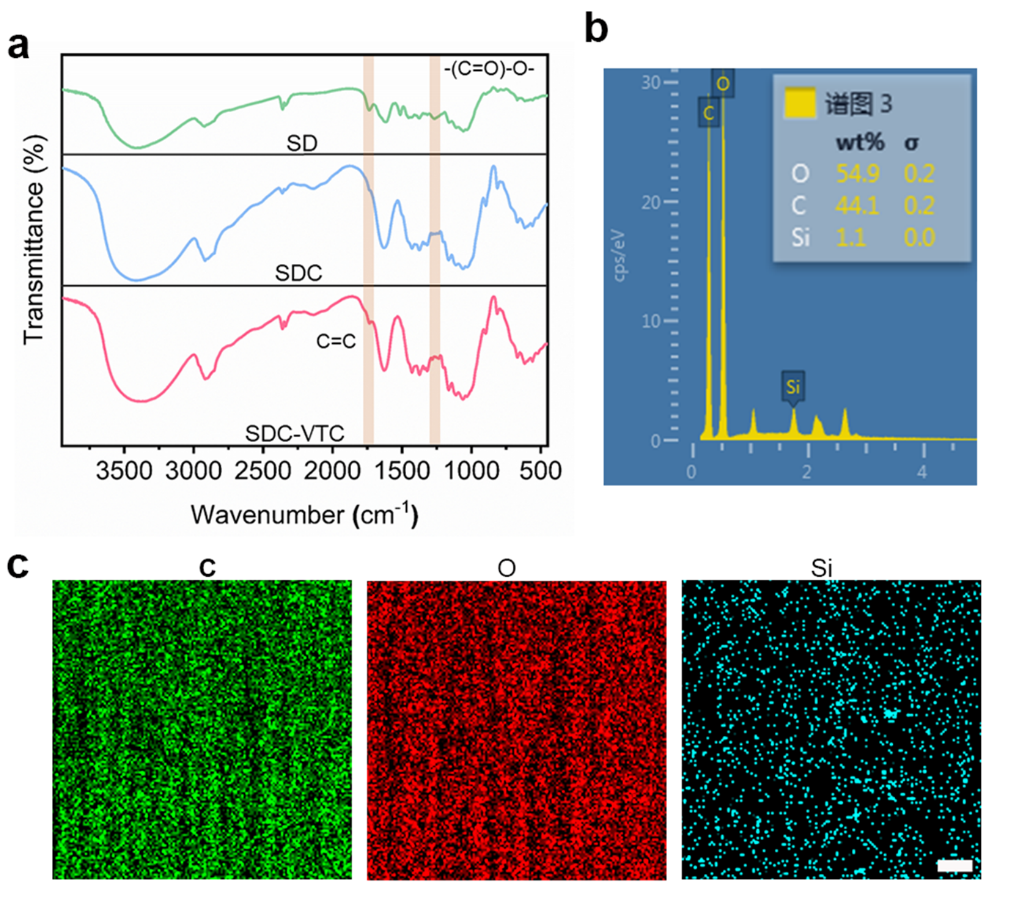


**Figure S2.** FTIR spectra and EDS mapping. (a) FTIR spectra of the SD, SDC and SDC-VTC. The SDC features a decrease in the peak intensities at 1735 cm^-1^ and 1260 cm^-1^, which respectively correspond to the carboxyl groups of hemicellulose and the ester linkage of the carboxyl groups of lignin and/or hemicellulose. The SDC-VTC shows a notable increase at 1735 cm^-1^, which is associated with the functional vinyl groups of the VTC. (b) EDS spectrum of SDC-VTC with weight concentration for C, O, and Si. (c) EDS elemental (C, O, and Si) mapping images of SDC-VTC. Scale bar: 40 μm.


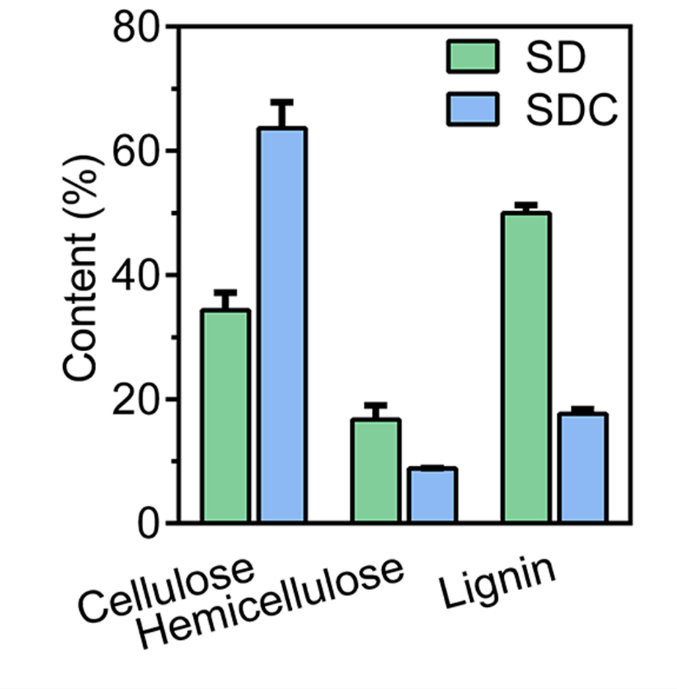


**Figure S3.** The ELISA results show that the evolution of the cellulose, hemicellulose, and lignin changed along with the delignification process from the SD to SDC. The results show that cellulose, hemicellulose, and lignin are partially removed by the chemical treatment. *n* = 5 independent samples.


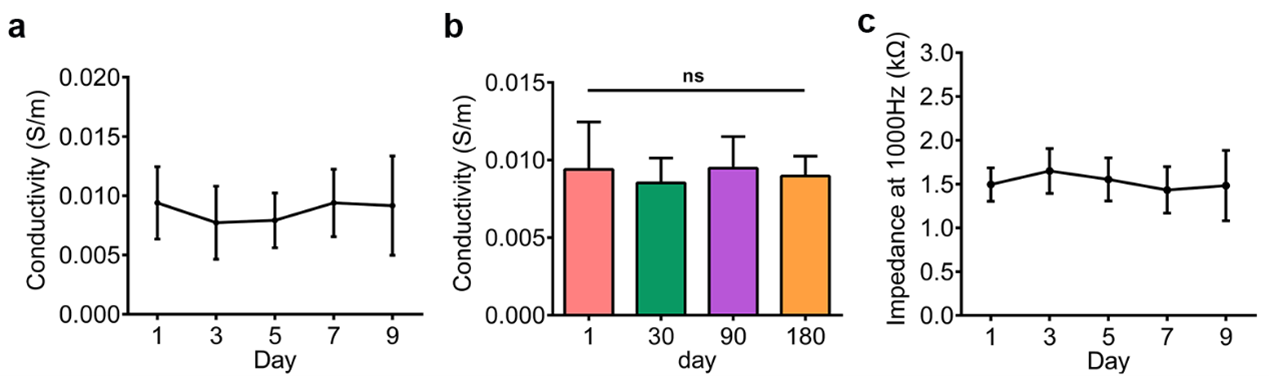


**Figure S4.** In vitro the electrical conductivity and EIS at 1000 Hz of the SDCHC in PBS at 37 °C. (a) Analysis of conductivity of the SDCHC on day 1, 3, 5, 7, 9. (b) Analysis of conductivity of the SDCHC on day 1, 30, 90, 180. Data are presented as mean ± S.D. statistical analysis (ns = no significance; p-value was generated by ANOVA and Tukey’s test; n = 5 independent samples). (c) Analysis of EIS of the SDCHC on day 1, 3, 5, 7, 9. n = 5 independent samples.


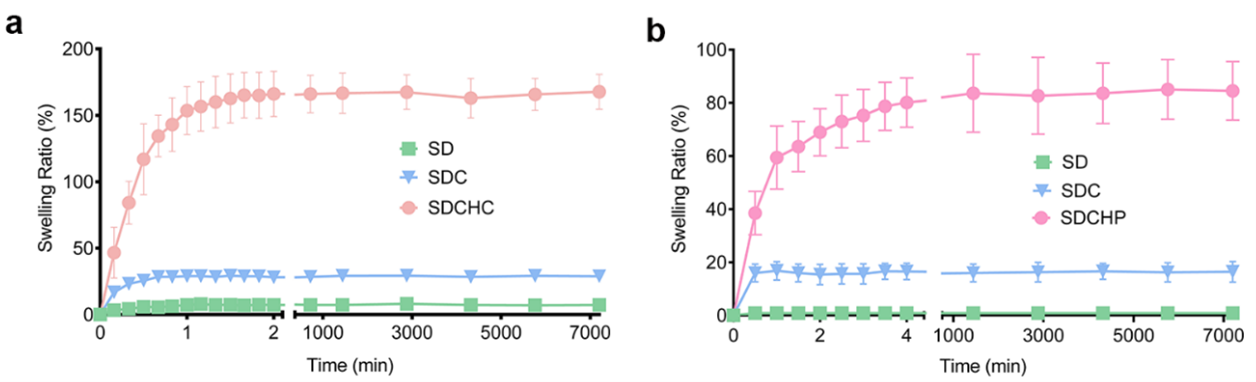


**Figure S5.** Analysis of swelling ratio of the SD, SDC, SDCHC and SDCHP in PBS (a) and deionized water (b) at 37 °C, respectively. *n* = 5 independent samples.


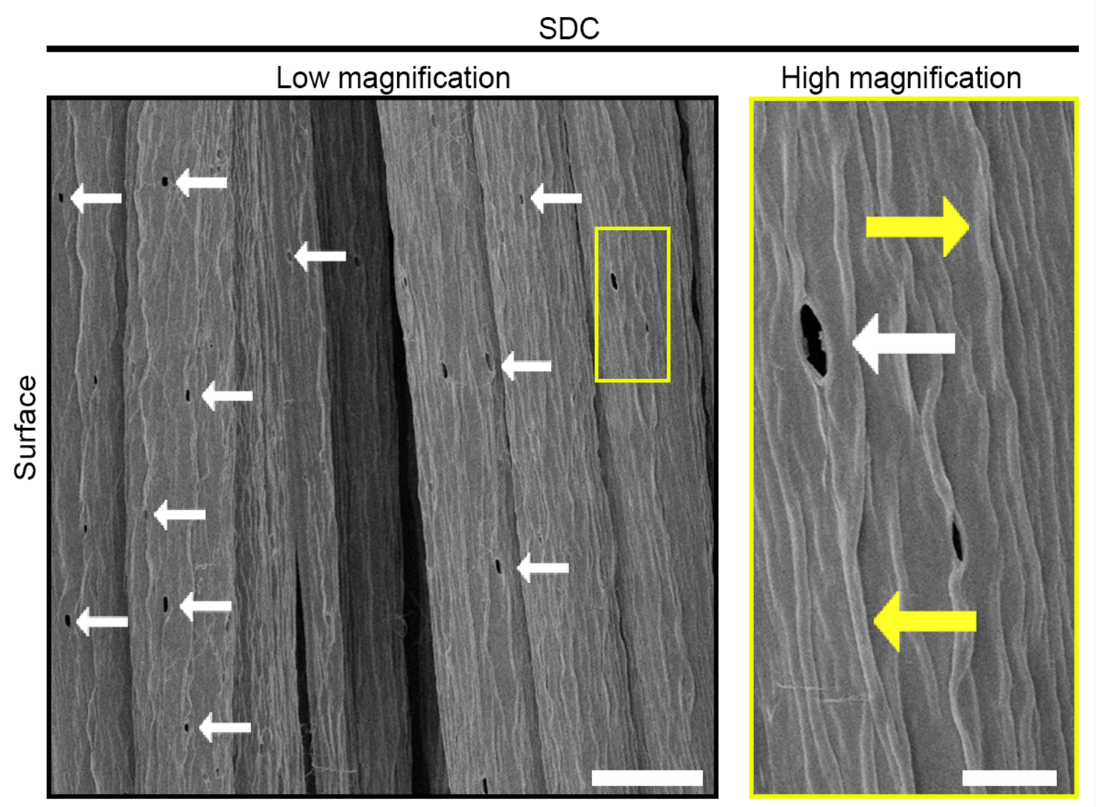


**Figure S6.** SEM image show the pore structure (white arrows) and submicrostructures (yellow arrows) on the surface of SDC. Scale bar: 20 μm at low magnification; 4 μm at high magnification.


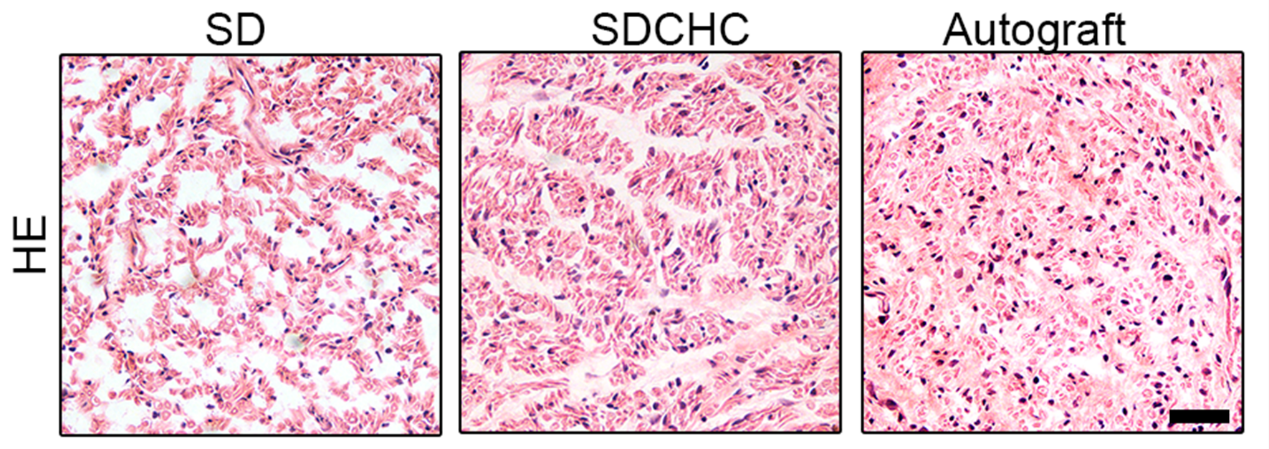


**Figure S7.** HE images in the central segment of regenerative nerve in the SD group, SDCHC group and autograft group at 16 weeks after surgery. Scale bars: 40 μm.


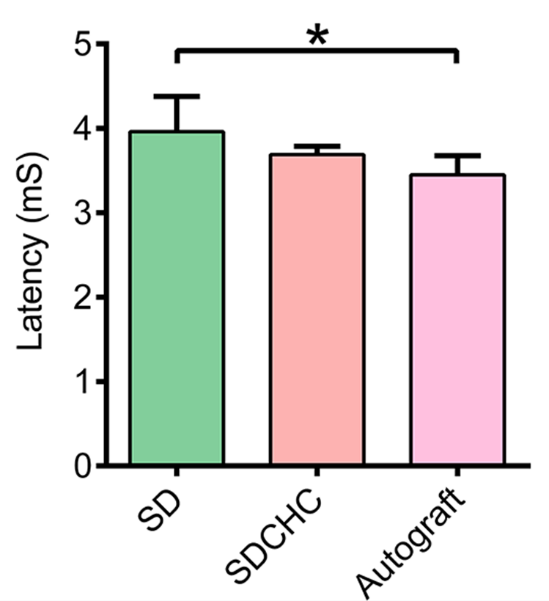


**Figure S8.** Quantitative analysis of latency at CMAP in SD group, SDCHC group and autograft group. (Data were presented as mean ± s.d. **p* < 0.05, ***p* < 0.01, ****p* < 0.001. *p* value was generated by ANOVA and Tukey’s test. *n* = 5 for SD group; *n* = 5 for SDCHC group; *n* = 5 for autograft group).


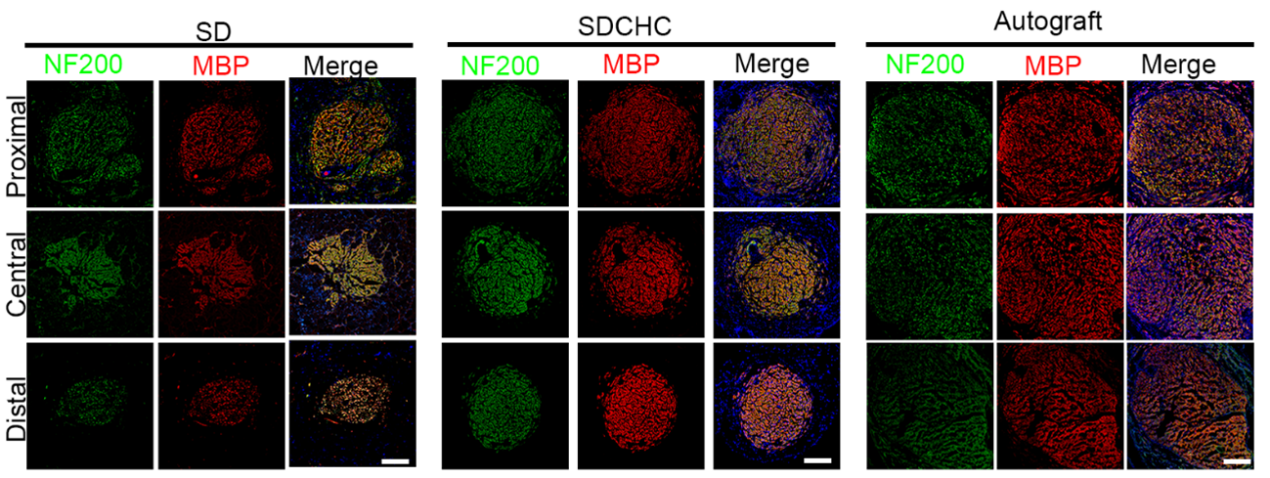


**Figure S9.** Immunostaining images of transverse sections of nerve fibers and myelin sheath in SD group, SDCHC group and autograft group at 16 weeks after surgery. Scale bars: 200 μm. Green represents nerve fiber; red represents myelin sheath; blue represents cell nucleus.


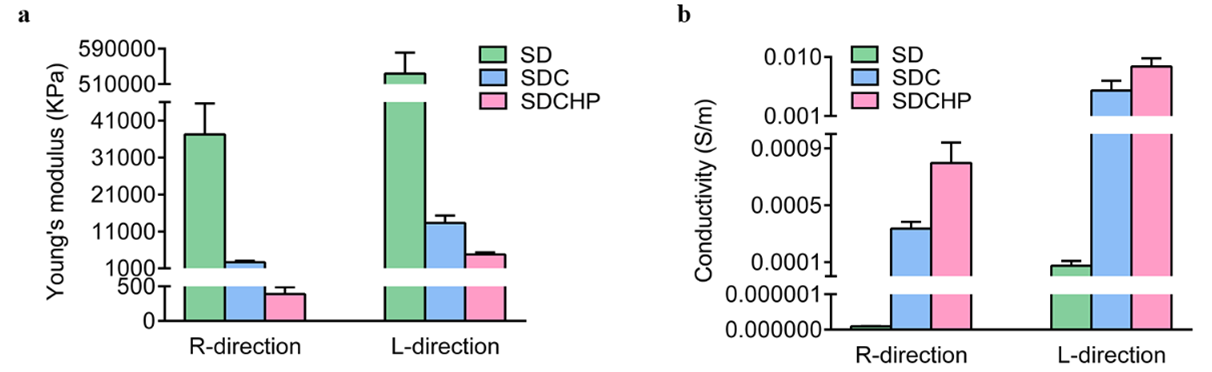


**Figure S10.** Analysis of electrical conductivity and Young’s modulus. (a) Analysis of mechanical of the SD, SDC, and SDCHP (n=3). (b) Analysis of electrical conductivity of the SD, SDC, and SDCHP (n=5). R-direction: Parallel of samples; L-direction: Vertical of samples.


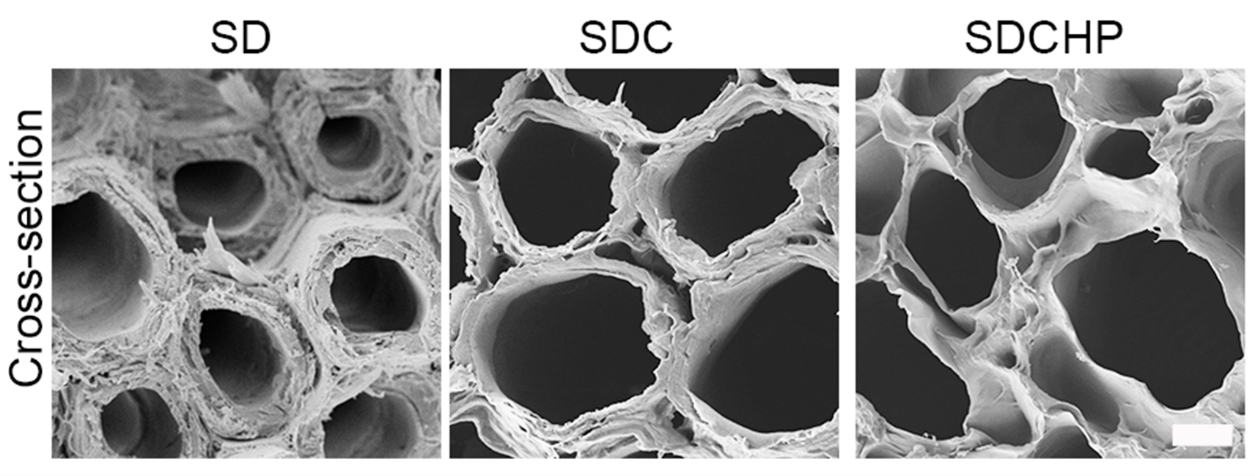


**Figure S11.** SEM image on the cross section of the SD, SDC and SDCHP. Scale bars: 10 μm.


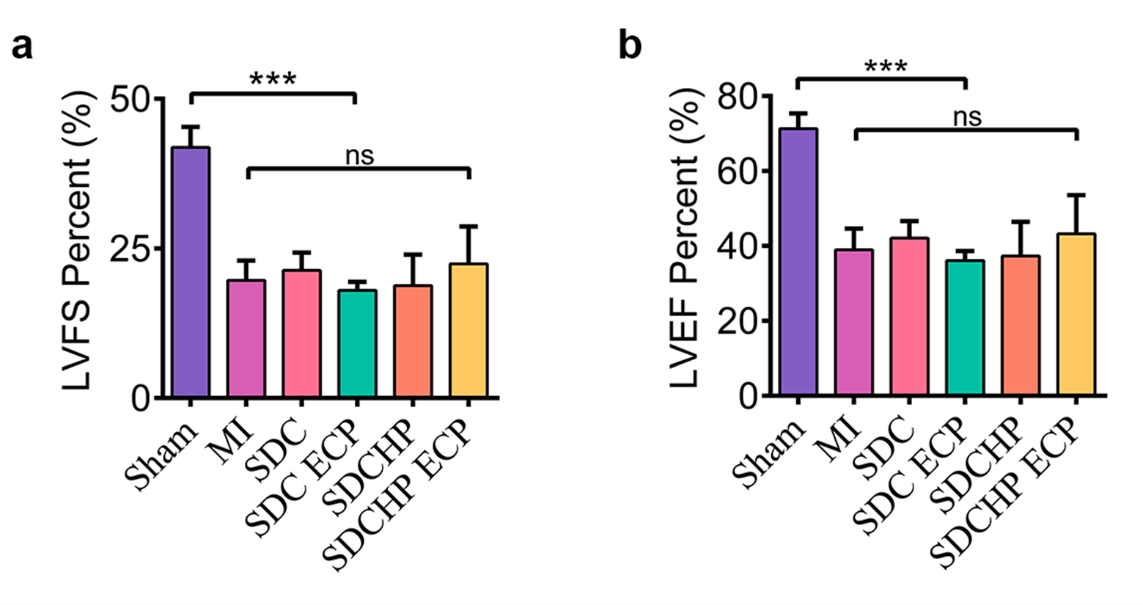


**Figure S12.** Analysis of the LVFS (a) and LVEF (b) at day 6 among each group. (Data were presented as mean ± s.d. **p* < 0.05, ***p* < 0.01, ****p* < 0.001. *p* value was generated by ANOVA and Tukey’s test. *n* = 3 for sham group; *n* = 5 for MI group; *n* = 5 for SDC group; *n* = 5 for SDC ECP group; *n* = 5 for SDCHP group; *n* = 5 for SDCHP ECP group).


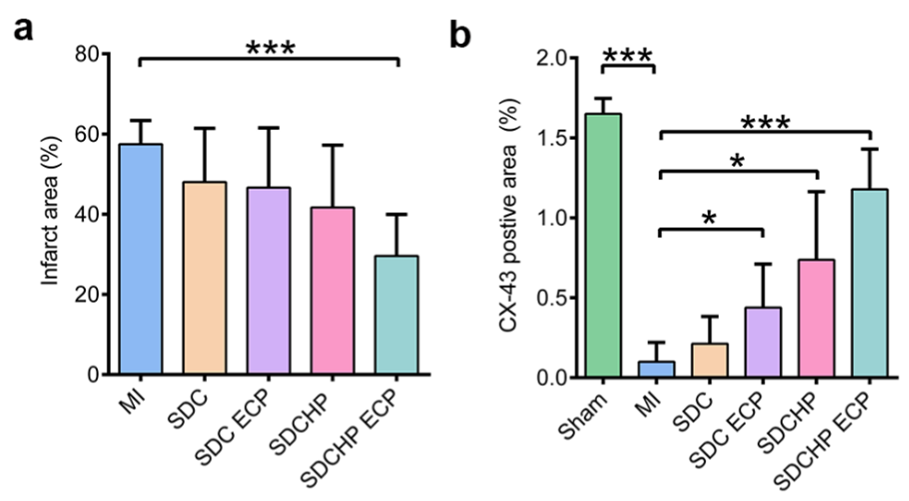


**Figure S13.** Quantitative analysis of (a) infarct area of left ventricular anterior wall and (b) CX-43 positive area based on the immunostaining images. (Data were presented as mean ± s.d. **p* < 0.05, ***p* < 0.01, ****p* < 0.001. *p* value was generated by ANOVA and Tukey’s test. *n* = 5 for SD group; *n* = 5 for SDCHC group; *n* = 5 for autograft group).
